# Self-Redirection of Metabolic Flux toward Squalene and Ethanol Pathways by Engineered Yeast

**DOI:** 10.3390/metabo10020056

**Published:** 2020-02-01

**Authors:** Robina Manzoor, Maqbool Ahmed, Naveeda Riaz, Bushra Hafeez Kiani, Ullah Kaleem, Yasmeen Rashid, Ali Nawaz, Muhammad Umer Farooq Awan, Hooria Khan, Umera Imtiaz, Yasir Rasheed, Imdad Kaleem, Aamir Rasool

**Affiliations:** 1 School of Life Science, Beijing Institute of Technology, Beijing 100081, China; 3820150009@bit.edu.cn; 2 Department of Tuberculosis, Bolan University of Medical and Health Sciences, Quetta 87300, Pakistan; maqboola93@gmail.com; 3 Department of Biological Sciences, International Islamic University, Islamabad 45550, Pakistan; Naveeda.riaz@iiu.edu.pk (N.R.); bushra.hafeez@iiu.edu.pk (B.H.K.); 4 Department of Microbiology, University of Balochistan, Quetta 87300, Pakistan; kaleem.mandokhail@gmail.com; 5 Department of Biochemistry, University of Karachi, Karachi 75530, Pakistan; Yrasid2004@yahoo.com; 6 Department of Industrial Biotechnology, Government College University, Lahore 53801, Pakistan; ali.nawaz@gcu.edu.pk (A.N.); umii.imtiaz@gmail.com (U.I.); 7 Department of Botany, Government College University, Lahore 53801, Pakistan; dr.umerfarooqawan@gcu.edu.pk; 8 Department of Bioscience, COMSATS Institute of Information Technology (CIIT), Islamabad 45550, Pakistan; hooriakhan.pk@gmail.com (H.K.); ginaaphoomichu40@gmail.com (Y.R.); 9 Institute for Synthetic Biosystem, School of Chemistry and Chemical Engineering, Beijing Institute of Technology, Beijing 100081, China; 10 Institute of Biochemistry, University of Balochistan, Quetta 87300, Pakistan

**Keywords:** *Saccharomyces cerevisiae*, engineered promoters, repressible promoters, squalene, fusel alcohol, ethanol

## Abstract

We have previously reported that squalene overproducing yeast self-downregulate the expression of the ethanol pathway (non-essential pathway) to divert the metabolic flux to the squalene pathway. In this study, the effect of co-production of squalene and ethanol on other non-essential pathways (fusel alcohol pathway, FA) of *Saccharomyces cerevisiae* was evaluated. However, before that, 13 constitutive promoters, like *IRA1p, PET9p, RHO1p, CMD1p, ATP16p, USA3p,* *RER2p, COQ1p, RIM1p, GRS1p, MAK5p,* and *BRN1p,* were engineered using transcription factor bindings sites from strong promoters *HHF2p* (−300 to −669 bp) and *TEF1p* (−300 to −579 bp), and employed to co-overexpress squalene and ethanol pathways in *S. cerevisiae.* The FSE strain overexpressing the key genes of the squalene pathway accumulated 56.20 mg/L squalene, a 16.43-fold higher than wild type strain (WS). The biogenesis of lipid droplets was stimulated by overexpressing *DGA1* and produced 106 mg/L squalene in the FSE strain. *AFT1p* and *CTR1p* repressible promoters were also characterized and employed to downregulate the expression of *ERG1,* which also enhanced the production of squalene in FSE strain up to 42.85- (148.67 mg/L) and 73.49-fold (255.11 mg/L) respectively. The FSE strain was further engineered by overexpressing the key genes of the ethanol pathway and produced 40.2 mg/mL ethanol in the FSE1 strain, 3.23-fold higher than the WS strain. The FSE1 strain also self-downregulated the expression of the FA pathway up to 73.9%, perhaps by downregulating the expression of *GCN4* by 2.24-fold. We demonstrate the successful tuning of the strength of yeast promoters and highest coproduction of squalene and ethanol in yeast, and present *GCN4* as a novel metabolic regulator that can be manipulated to divert the metabolic flux from the non-essential pathway to engineered pathways.

## 1. Introduction

Squalene is a polyunsaturated triterpenoid that acts as a precursor for the biosynthesis of steroids and cholesterol in animals and plants [1,2]. Several terpenoid synthases use squalene as a substrate to synthesize various types of triterpenoids including α-amyrin, β-amyrin, and lupeol [3,4]. Squalene is used in many cosmetic products because it acts as an emollient, antioxidant, and moisturizing agent [5,6]. Squalene has also been approved as a cardioprotective [7] and radioprotective agent [8].

The large proportion of squalene is extracted from the liver of sharks to fulfill the industrial demand [9]. However, the constant supply of the squalene is at risk due to the sharp decline in the number of sharks and international concern regarding the protection of marine wildlife [10,11]. *Saccharomyces cerevisiae* can accommodate squalene in the lipid droplets, microsome, and void spaces of the plasma membrane [12,13]. This makes it an ideal candidate for industrial-scale production of squalene. Earlier, our engineered *S. cerevisiae* produced ~304.16 mg/L squalene in the shake flask using terbinafine, an inhibitor of squalene epoxidase [14] and synergistically downregulated the expression of ethanol production pathway [15]. This indicates that yeast can self-redirect the metabolic flux from a non-essential pathway to an engineered pathway to alleviate the metabolic burden on pathways critical for its growth [15].

Ethanol also harbors many industrial applications, such as it is used in the preparation of a variety of beverages and consumed by the transport sector as a biofuel [16]. Yeast is also in practice for eight millennia for the production of a variety of alcoholic beverages [17]. Fermenting yeast not only excrete ethanol in the growth medium but also excrete several other low-molecular-weight compounds collectively termed as fusel alcohol [18]. Ethanol production is not necessary for the survival of yeast, because inhibition of its biosynthesis does not affect yeast growth [19,20]. *S. cerevisiae* produces fusel alcohol of different carbon chain length depending upon the type of substrates, such as it produces 2-Phenylethanol, tyrosol, and tryptophol through transamination and decarboxylation of phenylalanine, tyrosine, and tryptophan [18]. Yeast consumes one mole of NADH and excretes another mole of carbon for the production of one mole of ethanol, but it can re-metabolize ethanol in the subsequent growth phases [15]. On the other hand, yeast consumes one mole of ATP and NADH and excretes another one mole of CO_2_ for the production of one mole of fusel alcohol [18]. However, it cannot re-metabolize the excreted fusel alcohol to fulfill its energy requirements during the late exponential and stationary phases. A study has reported the overproduction of naringenin by deleting the byproduct forming genes including, *Aro10, Pdc5,* and *Pdc6* of fusel alcohol pathway without affecting the growth rate of engineered yeast [21]. This endorses our hypothesis that the fusel alcohol pathway is not-essential for *S. cerevisiae*, and it can be manipulated for redirection of metabolic flux to the engineered pathways the same as the ethanol production pathway.

In this study, *S. cerevisiae* was engineered with squalene and ethanol production pathways to determine their coordinated effect on the expression and production of the fusel alcohol pathway. Promoter engineering is a useful strategy adopted to optimize the expression of genes of the engineered pathway for the overproduction of high-value compounds [22]. Therefore, the strength of our earlier characterized 13 yeast constitutive promoters was tuned using transcription factor binding sites (TFBS) from strong promoters *HHF2p* (−300 to −669 bp) and *TEF1p* (−300 to −579 bp) and subsequently employed to overexpress the squalene and ethanol production pathways in *S. cerevisiae* [14].

Expression of target genes can be regulated by using repressible promoters and therefore often employed to control the expression of genes competing for precursors with engineered pathways [23]. In this study, two novel metal ion repressible promoters *AFT1p* (iron) and *CTR1p* (copper) were also characterized and used to downregulate the expression of *ERG1* for squalene overproduction in yeast.

**Figure 1:**
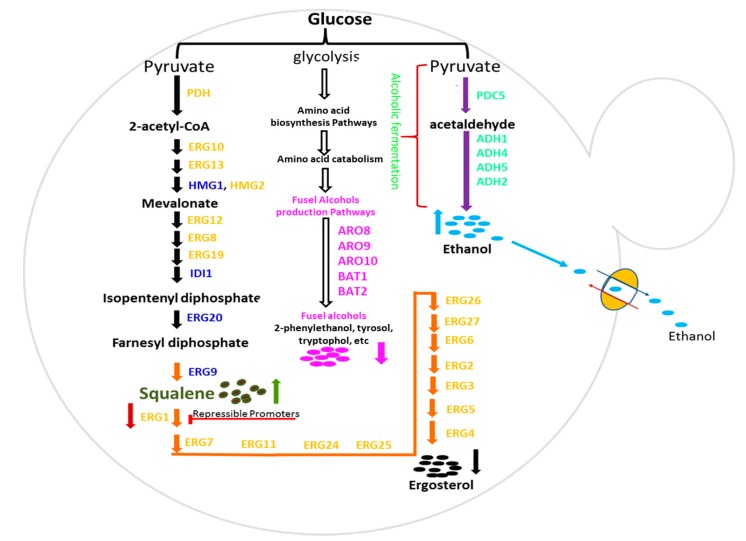
Engineering scheme of FSE1 strain: Engineered strain was constructed by co-overexpressing the vital genes of squalene and ethanol production pathways. Overexpressed genes of squalene and ethanol production pathways are written in blue and green, respectively. Auto-downregulated genes of the fusel alcohol production pathway are mentioned in purple.

Squalene and ethanol pathways were co-overexpressed in *Saccharomyces cerevisiae* using engineered constitutive promoters for squalene and ethanol co-production. Metal ion repressible promoters were used to optimize the production of squalene, and determined the synergistic effect of squalene and ethanol co-overproduction on the expression of fusel alcohol pathway (Figure 1). 

## 2. Results and Discussion

### 2.1. Tuning the Strength of Yeast Promoters

**Figure 2:**
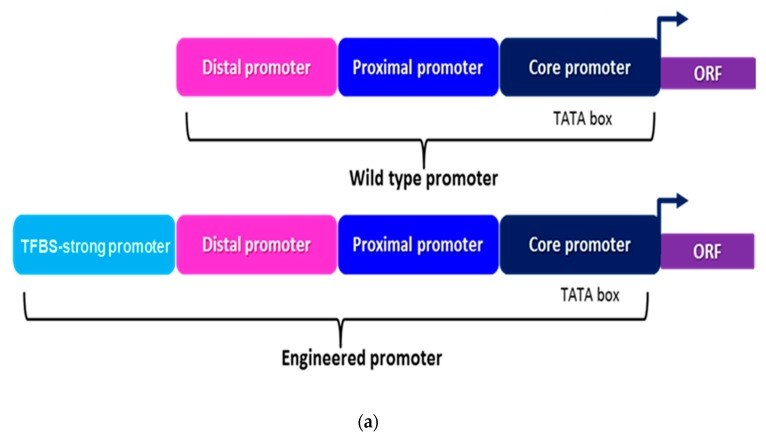

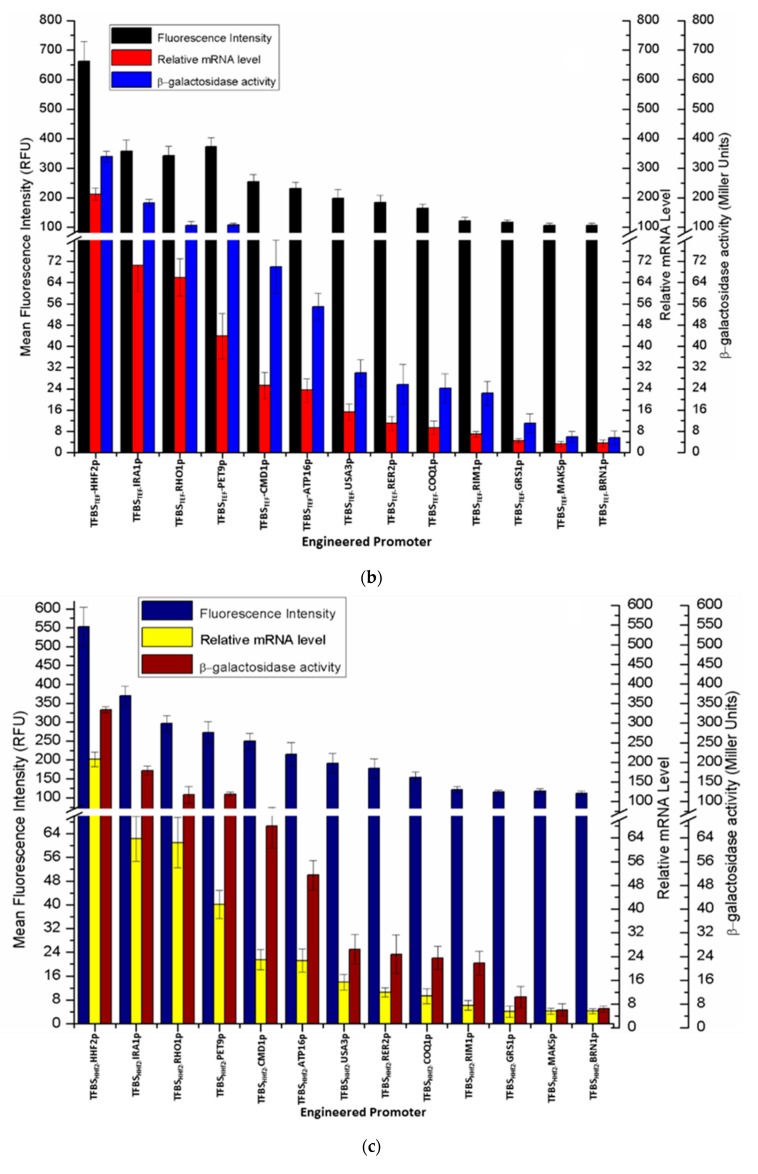

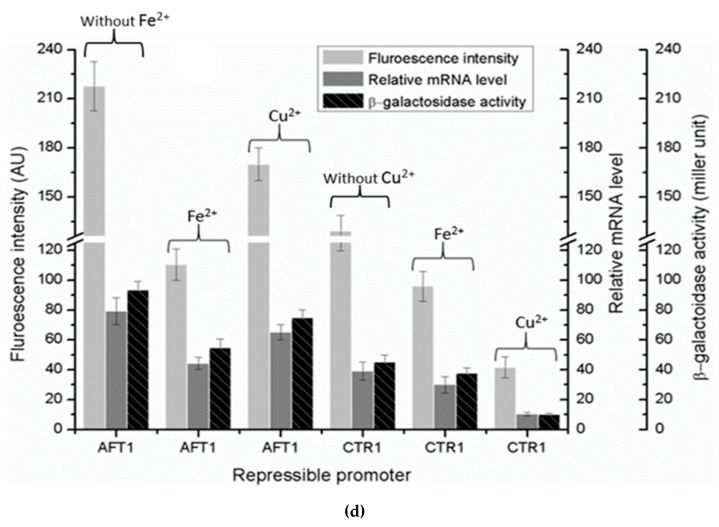
Tuning of the strength of yeast constitutive promoters: (**a**) Strategy for tuning the strength of yeast constitutive promoters transcription factor binding sites (TFBS) from strong constitutive promoters *TEF1p* (−300 to −579bp) and *HHF2p* (-300 to -669bp). (**b**) Characterization of the strength of engineered promoters constructed by ligating the TFBS_-*TEF1p*_ upstream of *HHF2p, IRA1p, RHO1p, PET9p, CMD1p, ATP16p, USA3p, RER2p, COQ1p, RIM1p, GRS1p, MAK5p,* and *BRN1p* using reporter genes *EGFP* and *LacZ*. (**c**) Characterization of the strength of engineered promoters constructed by ligating the TFBS_-*HHF2p*_ upstream of *HHF2p, IRA1p, RHO1p, PET9p, CMD1p, ATP16p, USA3p, RER2p, COQ1p, RIM1p, GRS1p, MAK5p,* and *BRN1p* using reporter genes *EGFP* and *LacZ*. Error bars represent the standard deviation of biological triplicate. (**d**) Characterization of the strength of metal ion repressible promoters. The strength of *AFT1p* and *CTR1p* was characterized using reporter genes *EGFP* and *LacZ*. Metals ion, Fe^2+^, and Cu^2+^, were tested to repress the strength of *AFT1p* and *CTR1p* promoters. Both metals ion repressed the strength of *AFT1p* and *CTR1p* to a different level. Error bars represent the standard deviation of biological triplicate.

Robust and balanced expression of the genes is a prerequisite for the successful overproduction of high-value compounds in engineered microbes [24,25]. This can be achieved using strong and well-characterized constitutive promoters capable of producing a higher titer of the enzymes robustly converting the substrate into the final product [26]. Although the repertoire of yeast constitutive promoters is large enough to engineer the medium size metabolic pathway,, this repertoire does not possess a large number of strong promoters [27,28,29]. Only a few studies have reported the successful use of weak promoters and low copy plasmids to optimize the expression of the pathway to produce high-end products including, the precursor of Taxol in *E. coli* [30]. Transcription factors control the strength of promoter by regulating the binding of RNA polymerase on the core promoter through binding at the transcription factor binding sites (TFBS) present upstream of the core promoter. Thus composition and hierarchy of TFBS upstream of the core promoter regulate the strength of the promoter [22]. The previous report has shown that strength of strong constitutive promoter *(GPDp)* and strong inducible promoters *(GALp)* can be further enhanced by adding the enhancer element sequences upstream of core promoter acting as synthetic transcriptional amplifiers [22]. In this study, the strength of previously characterized constitutive promoters is tuned by adding the TFBS from strong promoters (Figure 2a) [14]. Transcription factor binding sequences from *TEF1p* (−300 to −579 bp) and *HHF2p* (−300 to −669 bp) promoters were fused upstream of *HHF2p, IRA1p, RHO1p, PET9p, CMD1p, ATP16p, USA3p, RER2p, COQ1p, RIM1p, GRS1p, MAK5p* and *BRN1p* (Figure 2b,c, Table S6) and resulting promoters have been listed in the Table S6. The strength of all aforementioned promoters after ligating the TFBS was reckoned via measuring the fluorescence intensity and mRNA level of enhanced green fluorescent protein (EGFP) and catalytic activity of β-galactosidase.

The strength of *HHF2p, IRA1p, RHO1p, PET9p, CMD1p, ATP16p, USA3p, RER2p, COQ1p, RIM1p, GRS1p, MAK5p* and *BRN1p* was increased by 56.39%, 101.7%, 95.51%, 164.89%, 108.69%, 97.63%, 96.49%, 81.53%, 63.21%, 24.48%, 21.49%, 21.322%, and 27.45%, respectively after adding the TFBS_-TEF1_ (Figure 2b, Table S6). The strength of *HHF2p, IRA1p, RHO1p, PET9p, CMD1p, ATP16p, USA3p, RER2p, COQ1p, RIM1p, GRS1p, MAK5p* and *BRN1p* was enhanced by 36.88%, 59.69%, 51.43%, 100.42%, 92.52%, 85.74%, 76.36%, 80.75%, 48.53%, 26.467%, 23.89%, 42.46%, and 50.57%, respectively after attaching the TFBS_-HHF2_ (Figure 2c, Table S6). The strength of all promoters was enhanced to a different level by adding the TFBS of *TEF1p* and *HHF2p* (Figure 2b,c, Figure S5a–c). The increase above in the strength of engineered promoters was measured using an increase in fluorescence intensity of EGFP compared to the wild type promoters (Table S5). TFBS upstream of wild type promoters did not inflict adverse effect on the constitutiveness of wild type promoters, and this is also evident from the strong the induction of fluorescent intensity, mRNA level of EGFP and catalytic activity of β-galactosidase without an inducer by engineered promoters compared to the wild type promoters (Figure 2b,c and Figure S5a–c). Yeast cells expressing *EGFP* downstream of engineered promoters are brighter than yeast cells expressing *EGFP* downstream of wild type promoters during confocal microscopy analysis keeping the analysis time fixed to 100 ms for each sample (Figure S5a–c).

The results of this study indicate that the strength of yeast constitutive promoters can be enhanced using TFBS of strong promoters. Moreover, the strength of the promoters can also be in-situ tuned by using multiplex gRNA carrying TFBS in the CRISPR-Cas9 system [31].

### 2.2. Characterization of Metal Ion Repressible Promoters

Repressible promoters, a vital tool of metabolic engineering, are employed to downregulate the expression of genes competing for precursors with an engineered pathway. Methionine repressible promoter *(Met3p)* has been previously employed to downregulate the expression of different genes [32], but its use is not industrially viable due to its high cost and consumption by the cell for growth.

Metal ion repressible promoters are a sustainable alternative to metabolite repressible promoters due to their cheap cost and little consumption by the cell for growth. Transcription factor Aft1 induces the expression of iron regulon genes in the iron-deficient yeast cell and promotes the iron uptake by stimulating the remodeling of cellular metabolism [33]. *CTR1* encodes plasma membrane protein required for high-affinity uptake of copper from the growth medium, but its transcription is strongly repressed by copper ions [34]. The repression of *AFT1p* and *CTR1p* was determined in terms of fluorescence intensity and relative mRNA level of *EGFP* and unit activity of β-galactosidase. The strength of *AFT1p* was repressed by 2-fold and 1.33-fold, respectively, using Fe^2+^ and Cu^2+^ (Figure 2d). The strength of *CTR1p* was repressed by 1.42-fold 3.38-fold, respectively, using Fe^2+^ and Cu^2+^ (Figure 2d).

### 2.3. Overexpression of the Squalene Synthesis Pathway in Yeast Via Engineered Promoters

**Figure 3:**
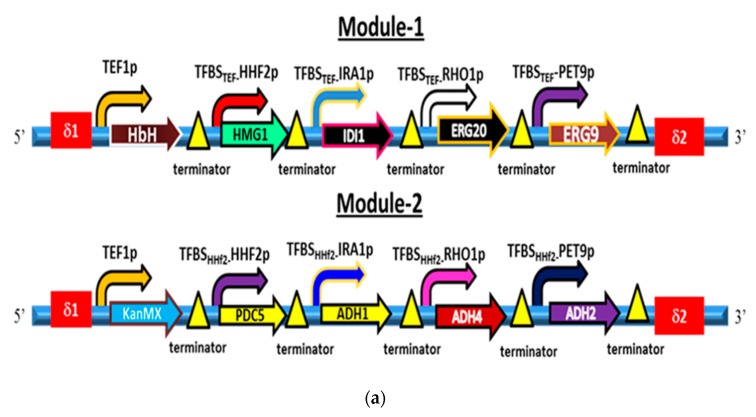

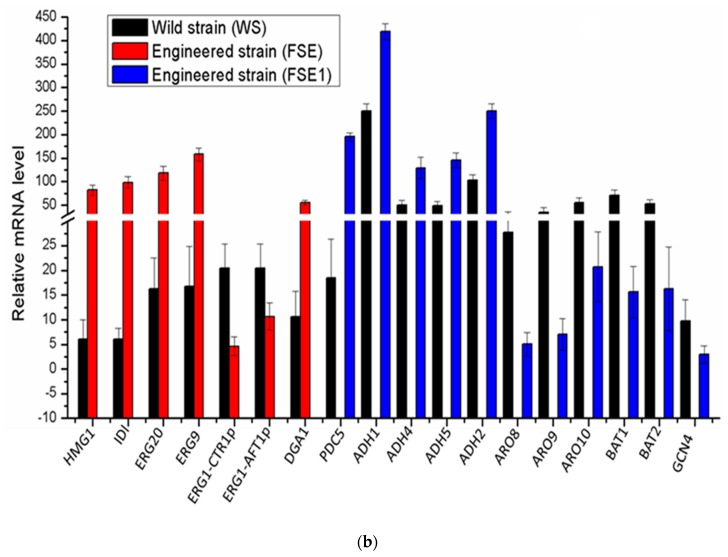
(**a**) Module-1 consists of *HMG1, IDI1, ERG20* and *ERG9* genes of squalene biosynthesis pathway, was transformed into *Saccharomyces cerevisiae* leading to the FSE strain and module-2 comprises of *PDC5, ADH1, ADH4, ADH5* and *ADH2* genes of ethanol production pathway was transformed into FSE strain leading to the FSE strain. Engineered promoters *TFBS_TEF_-HHF2p, TFBS_TEF_-IRA1p, TFBS_TEF_-RHO1p, TFBS_TEF_-PET9p,* and *TFBS_HHF2_-HHF2p, TFBS_HHF2_-IRA1p, TFBS_HHF2_-RHO1p, TFBS_HHF2_-PET9p* were employed to overexpress the genes of squalene and ethanol production pathways, respectively. Names of the terminators are given in Table S2 with their primers. (**b**) qRT-PCR analysis of (i) overexpressed genes of SB and EP pathways, (ii) *ERG1* under repressible promoters, (iii) genes of fusel alcohol pathway: The expression level of *HMG1*, *IDI1*, *ERG20* and *ERG9* genes of squalene biosynthesis pathway was enhanced in FSE strain compared to the WS after employing *TFBS_TEF1-_HHF2p, TFBS_TEF1-_IRA1p, TFBS_TEF1-_RHO1p,* and *TFBS_TEF1-_PET9p* promoters, respectively. The expression level of the *ERG1* gene was decreased in FSE strain compared to the WS after employing *AFT1p* and *CTR1p* repressible promoters. The expression level of *PDC5, ADH1*, *ADH4*, *ADH5,* and *ADH2* genes was enhanced in FSE1 strain compared to the WS after employing the *TFBS_HHF2-_HHF2p, TFBS_HHF2-_IRA1p, TFBS_HHF2-_RHO1p,* and *TFBS_HHF2-_PET9p* promoters, respectively. Engineered promoters enhanced the expression of target genes of each pathway, and metal ion repressible promoters downregulated the expression of *ERG1* genes. The expression level of genes such as transcription factor regulator *GCN4* and *ARO8*, *ARO9, ARO10, BAT1*, and *BAT2* of fusel alcohol pathway in FSE1 strain compared to the wild type strain (WS) was downregulated. Error bars represent the standard deviation of biological triplicate.

**Figure 4:**
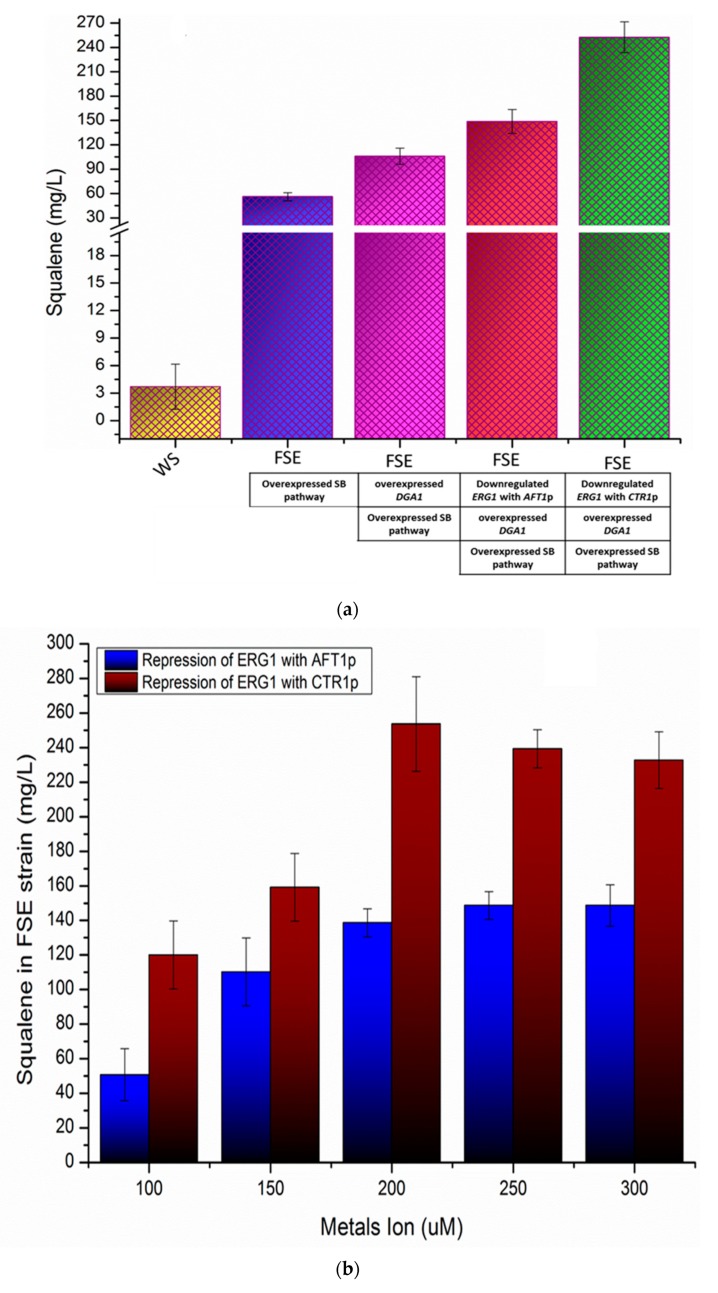

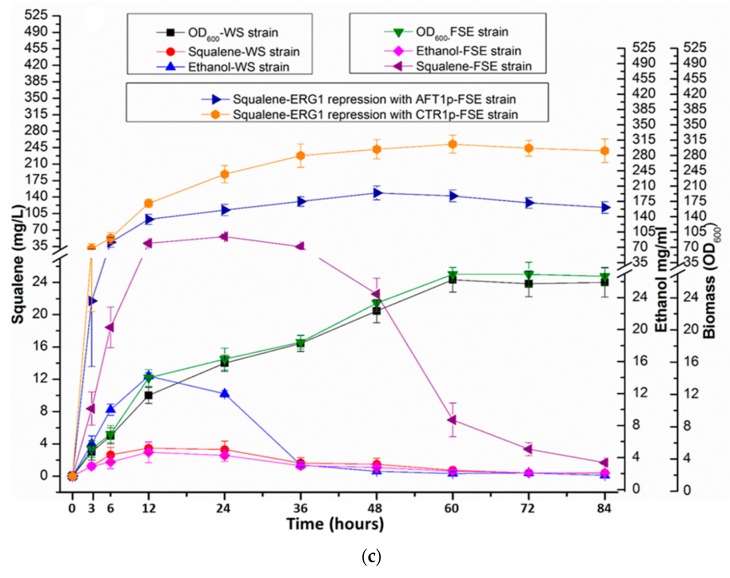
(**a**) Optimization of squalene overproduction in FSE strain. Production of squalene in FSE strain was optimized by overexpressing the *DGA1* and downregulation of *ERG1* using *AFT1p* and *CTR1p* repressible promoters. By overexpressing *DGA1* and downregulation of *ERG1* using *CTR1p* maximum amount of squalene was produced in FSE strain. Error bars represent the standard deviation of biological triplicate. **(b)** Dose optimization of metals ion for the downregulation of *ERG1* to optimize the squalene production in FSE strain. Different concentrations of Fe^2+^ and Cu^2+^ including, 100 uM, 150 uM, 200 uM, 250 uM, and 300 uM were tested to repress the *AFT1p* and *CTR1p* respectively. *CTR1p* was strongly repressed by Cu^2+^ at 200 uM, while *AFT1p* was strongly repressed by Fe^2+^ at 250 uM. The expression of *ERG1* was strongly downregulated using *CTR1p* at 200 uM of Cu^2+^ compared to the *AFT1p* at 250 uM of Fe^2+^. Error bars represent the standard deviation of biological triplicate. **(c)** Time course of squalene and ethanol co-production in FSE strain. Engineered FSE strain produced the maximum amount of squalene till 24 h of growth and started consuming it after 24 h of fermentation. The downregulation of *ERG*1 by *AFT1p* and *CTR1p* separately caused the maximum accumulation of squalene up to 48 h and 60 h, respectively, in FSE strain. FSE strain producing maximum titer of squalene by repressing *ERG1* with *CTR1p* also reduced the production of ethanol.

The terpenoid biosynthesis pathway of *S. cerevisiae* has been abundantly maneuvered for overproduction of heterologous isoprenoids, such as miltiradiene [35], artemisinic acid [36], α-santalene [37], β-amyrin [38] violacein [39]. Similarly, *S. cerevisiae* can also be customized to overproduce the native isoprenoids, including isopentenol, farnesol, and squalene, etc. Mevalonate pathway (MVA) is an indispensable segment of the squalene biosynthesis pathway (SB pathway), leading to the production of isopentenyl diphosphate precursor of squalene [40]. HMG-CoA (*HMG1*) reductase catalyzes the rate-limiting step of the MVA pathway; its overexpression has been reported to enhance the production of squalene in *S. cerevisiae* [41]. Isopentenyl diphosphate isomerase-1 (*IDI1*) controls the flow of isopentenyl pyrophosphate/dimethylallyl pyrophosphate (IPP/DMAPP) to the SB pathway [38]. Farnesyl diphosphate synthase (*ERG20*) stands at the major intersection of the SB pathway and synthesizes the FDP by condensing isopentenyl diphosphate that is used for biosynthesis of squalene, sterols, dolichols, ubiquinone and prenylated proteins [42]. Squalene synthase (*ERG9*) performs the first committed reaction of the ergosterol biosynthesis pathway leading to the production of squalene through condensation of two molecules of FDP. Keeping in view the critical role of HMG-CoA reductase, isopentenyl diphosphate isomerase-1, farnesyl diphosphate synthase, and squalene synthase in the biosynthesis of squalene, the genes *HMG1, IDI1, ERG20,* and *ERG9* of enzymes mentioned above were overexpressed using *TFBS_TEF-_HHF2p, TFBS_TEF-_IRA1p, TFBS_TEF-_RHO1p,* and *TFBS_TEF-_PET9p* respectively (Figure 3a). The expression of *HMG1, IDI1, ERG20,* and *ERG9* was enhanced by 13.67-fold, 16.33-fold, 7.24-fold, and 9.46-fold, respectively in FSE strain than WS (Figure 3b). FSE strain also produced 16.43-fold (56.20 mg/L) higher squalene than WS (Figure 4a). The production of squalene in FSE strain was confirmed through GC-MS spectrometry by comparing the GC-MS spectra of the sample with an authentic standard of squalene and fragmentation fingerprint of squalene in the NIST library database (Figure S6a,e). The overproduction of squalene in FSE strain also caused the reduction of ethanol production in FSE strain by 4.18 fold than the WS strain (Figure 4c) and endorsed the results of the earlier study [15].

### 2.4. Optimization of Squalene Overproduction Via Overexpression of DGA1 and Downregulation of ERG1

The excessive cytoplasmic accumulation of olefin oils such as squalene and terpene inflict toxic effects and metabolic stress on the yeast cell [43,44]. However, lipid droplets in yeast can only store small quantities of olefin oil due to the tight regulation of their biogenesis [45,46]. The main function of lipid droplets is to store the hydrophobic olefins oils and relieve their toxic effect on yeast cells [47,48].

*DGA1* encodes diacylglycerol O-acyltransferase, catalyzes the critical step involved in the production of triacylglycerol (TAG) and stimulation of biogenesis of lipid droplets in yeast [49]. The previous study has shown that overexpression of *DGA1* enhanced the accumulation of squalene via stimulating the biogenesis of lipid droplets in yeast [44].

In this study, expression of *DGA1* was enhanced by 5.25 fold using the TFBS-_TEF1_IRA1 promoter, leading to the accumulation of squalene in FSE strain up to 106 mg/L, a 28.61-fold higher than WS strain (Figure 3b and Figure 4a). The biogenesis of lipid droplets in the FSE1 strain was also enhanced due to the overexpression of *DGA1* and visualized through staining with Nile red dye Figure S4 [50].

Squalene monooxygenase (*ERG1*) catalyzes the conversion of squalene into the squalene epoxide [51]. The downregulation of *ERG1* up to 1.92 fold and 4.45 fold by *AFT1p* and *CTR1p* respectively (Figure 3), enhanced the production of squalene up to 42.85-fold (148.67 mg/L) and 68.25-fold (252.51mg/L) respectively, in FSE strain higher than wild type strain (Figure 4a–c). It is evident from Figure 3b and Figure 4a–c that *CTR1p* strongly repressed the expression of *ERG1* compared to the *AFT1p* and caused the highest accumulation of squalene in FSE strain (Figure 4a–c). The addition of Cu^2+^ more than 200 uM reduced the growth rate of engineered strain and also reduced the production of squalene in FSE strain (Figure 4a), therefore in subsequent observations, 200 uM of Cu^2+^ was used.

To our knowledge, FSE strain produced the highest titer of the squalene in shake flask without using expensive inhibitors or repressors. The fermentation in the fed-batch fermenter can further enhance the titer of squalene by FSE strain.

### 2.5. Co-Overproduction of Ethanol in S. cerevisiae

Ethanol is a viable alternative to fossil fuel and also used in the preparation of various alcoholic beverages. Earlier it has been reported that squalene overproducing yeast self-downregulates the expression of ethanol production pathway (EP), a non-essential pathway for yeast growth and survival [15]. Later, through the literature review, it was also found that the fusel alcohol pathway is also non-essential for yeast growth and survival [18,22]. Therefore, ethanol and squalene production pathways were co-expressed to determine their impact on the expression and production of the fusel alcohol pathway in yeast.

**Figure 5:**
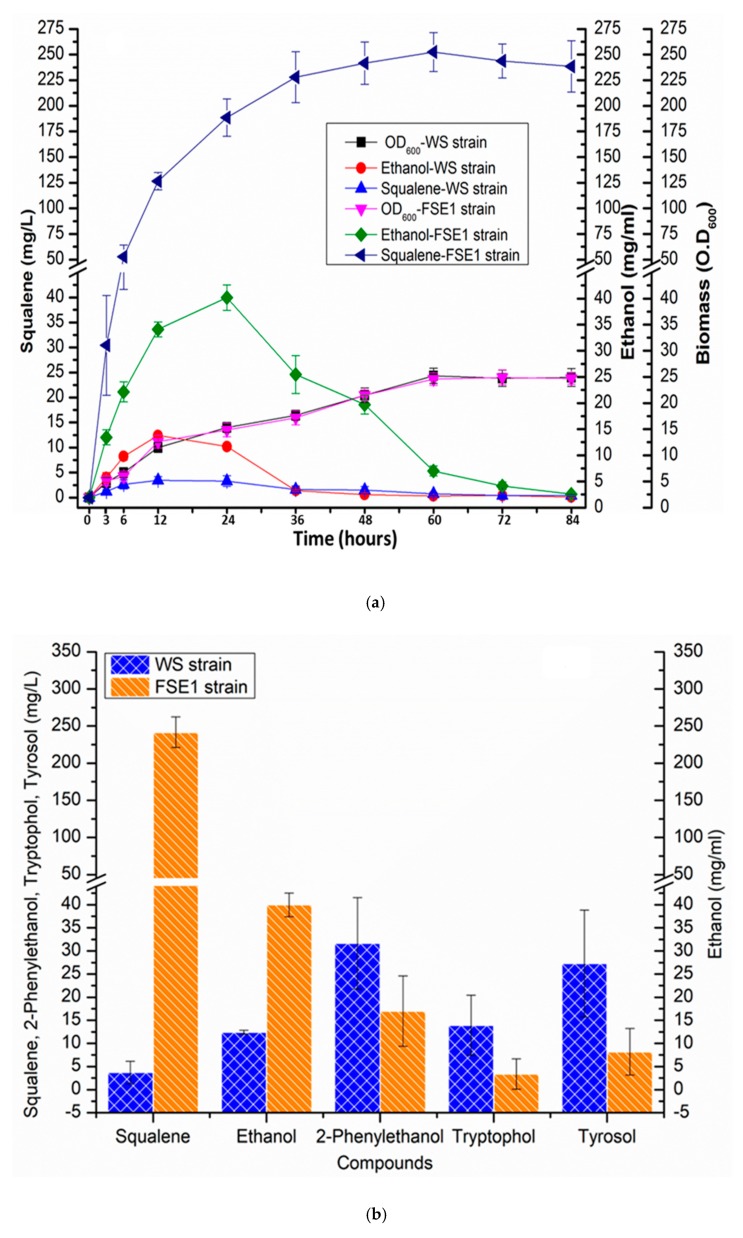
(**a**) Time course of squalene and ethanol co-production by FSE1 strain: Engineered FSE1 strain after co-expressing the squalene and ethanol pathways produced maximum titer of ethanol and squalene till 24th- and 60th hours respectively, of growth **(b)** Squalene and ethanol co-production in FSE1 strain caused the reduction of production of fusel alcohols including, 2-Phenylethanol, tyrosol, tryptophol compared to the WS. However, it also reduced the production of fusel alcohol including, 2-Phenylethanol, tryptophol, and tyrosol. It shows the self-diversion of metabolic flux by FSE1 in response to metabolic burden.

Pyruvate decarboxylase (PDC5) is a key enzyme of the ethanol pathway, catalyzes the conversion of pyruvate into acetaldehyde [52]. Alcohol dehydrogenase 1 (*ADH1*), alcohol dehydrogenase IV (*ADH4*), alcohol dehydrogenase 2 (*ADH2*), and alcohol dehydrogenase 5 (*ADH5*) catalyze the reduction of acetaldehyde into ethanol [53,54]. Essential genes, (i) *PDC5* (pyruvate decarboxylase 5)*,* (ii) *ADH1* (alcohol dehydrogenase 1), (iii) *ADH4* (alcohol dehydrogenase 4), (iv) *ADH5* (alcohol dehydrogenase 5) and (v) *ADH2* (alcohol dehydrogenase 2) of ethanol pathway using *TFBS_HHF2-_HHF2p, TFBS_HHF2-_IRA1p, TFBS_HHF2-_RHO1p,* and *TFBS_HHF2-_PET9p*, respectively were expressed in FSE strain leading to FSE1 strain (Figure 3a). Employment of engineered promoters successfully enhanced the expression of *PDC5* (pyruvate decarboxylase 5)*, ADH1* (alcohol dehydrogenase 1), *ADH4* (alcohol dehydrogenase 4), *ADH5* (alcohol dehydrogenase 5) and *ADH2* (alcohol dehydrogenase 2) by 10.56-, 1.68-, 2.58-, 2.97-, and 2.44-fold, respectively in FSE1 strain than wild type strain (Figure 3b). FSE1 strain co-overexpressing ethanol and squalene pathways co-produced 40.2 mg/mL and 255.11 mg/L ethanol and squalene, respectively (Figure 5a,b).

### 2.6. Yeast Co-Producing the Squalene and Ethanol Self-Downregulated the Expression of Fusel Alcohol Pathway

Fusel alcohols are produced by yeast during fermentation and impart flavor to fermented foods and beverages [17]. Fusel alcohol are produced from catabolism of amino acids [18], including branched-chain amino acids (leucine, valine, and isoleucine), aromatic amino acids (phenylalanine, tyrosine, and tryptophan), and sulfur-containing amino acid (methionine) [18].

Yeast tightly regulates the expression of anabolic and catabolic pathways through multiple regulatory networks [55]. The yeast SPT10 gene encodes a putative histone acetyltransferase (HAT) is implicated as a global transcription regulator acting through basal promoters [56]. In another study, the secretion of the SW14 protein was enhanced through global level tuning of gene expression by engineering the expression of TF. Earlier it has been reported that squalene overproducing yeast self-downregulated the ethanol production pathway through cryptic regulatory pathway [15]. Herein, it was aimed to evaluate the effect of squalene and ethanol co-overproduction on the expression of the fusel pathway of yeast.

It was found that squalene and ethanol co-overproducing FSE1 strain self-downregulated the expression of *ARO8* (aromatic/aminoadipate aminotransferase 1), *ARO9* (aromatic amino acid aminotransferase 2)*, ARO10* (transaminated amino acid decarboxylase)*, BAT1* (branched-chain amino-acid aminotransferase, mitochondria), and *BAT2* (branched-chain amino acid aminotransferase, cytosol) genes of fusel alcohol pathway by 5.48-fold, 5.05-fold, 2.67-fold, 4.54-fold, and 3.26-fold respectively, collectively 73.9%, than WS strain (Figure 3). Although fusel alcohol are comprised of catabolic products of branched-chain amino acids and aromatic amino acids, the effect of squalene and ethanol, co-overproduction was determined on the catabolism of aromatic amino acids, including, 2-phenylethanol (phenylalanine), tyrosol (tyrosine), tryptophol (tryptophan). FSE1 strain produced 2-phenylethanol, tyrosol, tryptophol by 1.87-fold, 4.12-fold and 3.33-fold respectively, less than WS strain (Figure 5b and Figure S6b–d,f–h). The expression of positive transcriptional factor regulator *GCN4* was also downregulated by 2.24-fold in the FSE1 strain (Figure 3). However, the effect of squalene overproduction was also determined on the fusel alcohol pathway, but no perturbation was observed. However, the production of ethanol was reduced in FSE strain by 4.18 fold than WS strain (Figure 4c), which was later improved in FSE1 strain by overexpressing the ethanol pathway (Figure 5a,b).

This indicates the presence of a cryptic global regulatory pathway operating through different regulators to redirect the metabolic flux to engineered pathways. We surmise that perhaps cryptic global regulatory pathway signaled the downregulation of *GCN4* and prevented the loss of carbon skeleton in the cell medium and directed the redirection of metabolic flux toward the engineered pathway. General amino acid control protein (*GCN4*), generally derepress amino acid biosynthesis pathways during starvation of amino acids and also plays a critical role in controlling the spatial organization of yeast genome [57,58]. *GCN4* positively regulates the transcription of target genes via binding at 5’-TGA[CG]TCA-3’ sequence in the promoters [57].

To our knowledge, this is the first report on the highest co-production of squalene and ethanol in yeast and demonstrating the self-diversion of metabolic flux from the fusel alcohol pathway toward engineered pathways.

## 3. Materials and Methods

### 3.1. Strains, Media, and Cells Cultivation

*Saccharomyces cerevisiae* INVSc1 (*MATa his3Δ1 leu2 trp1-289 ura3-52/MATa his3Δ1 leu2 trp1-289 ura3-52*) (Invitrogen, Carlsbad, CA) was manipulated to overexpress the squalene biosynthesis (SB) pathway and ethanol production (EP) pathways for squalene and ethanol co-production. Positive transformants harboring SB and EP pathways were selected on YPD agar plates (glucose 20 g/L, tryptone 20 g/L, yeast extract 10 g/L, agar 17 g/L) containing 500 mg/L hygromycin B (Roche, Indianapolis, IN) and 300 mg/L geneticin respectively. Positive transformants harboring *AFT1p* and *CTR1p* modules were selected on histidine negative nutrient agar plates, while positive transformants carrying *GCN4* module were selected on tryptophan negative nutrient agar plate. The optical density (OD_600_) of fermenting cultures was measured using a spectrophotometer (model U-2900, HITACHI, Chiyoda, Tokyo).

### 3.2. Tuning the Strength of Promoters

Engineered promoters named as TFBS_TEF1-_HHF2p, TFBS_TEF1-_IRA1p, TFBS_TEF1-_RHO1p, TFBS_TEF1-_PET9p, TFBS_TEF1-_CMD1p, TFBS_TEF1-_ATP16p, TFBS_TEF1-_USA3p, TFBS_TEF1-_RER2p, TFBS_TEF1-_COQ1p, TFBS_TEF1-_RIM1p, TFBS_TEF1-_GRS1p, TFBS_TEF1-_MAK5p, TFBS_TEF1-_BRN1p and TFBS_HHF2-_HHF2p, TFBS_HHF2-_IRA1p, TFBS_HHF2-_RHO1p, TFBS_HHF2-_PET9p, TFBS_HHF2-_CMD1p, TFBS_HHF2-_ATP16p, TFBS_HHF2-_USA3p, TFBS_HHF2-_RER2p, TFBS_HHF2-_COQ1p, TFBS_HHF2-_RIM1p, TFBS_HHF2-_GRS1p, TFBS_HHF2-_MAK5p, TFBS_HHF2-_BRN1p were constructed by attaching the TFBS of TEF1p (-300 to -579 bp) and HHF2p (-300 to -669 bp) promoters, respectively, upstream of HHF2p, IRA1p, RHO1p, PET9p, CMD1p, ATP16p, USA3p, RER2p, COQ1p, RIM1p, GRS1p, MAK5p, and BRN1p yeast constitutive promoters. All sequences of the promoters were amplified from the genomic DNA of S. cerevisiae. The reporter genes of EGFP and LacZ used to measure the strength of engineered promoters were amplified from previously constructed plasmid pET-28a (+)-pykA-egfp and genomic DNA of E. coli, respectively.

The primers for amplification of transcriptional factor binding sites, native promoters, and reporter genes were designed as that the 39 bp at 5′ end of each transcriptional binding site overlaps with 39 bp at 3′ end of the BamHI digested plasmid pRS41H, whereas 39 bp at 3′ end of each transcriptional binding site overlaps with 39 bp at 5′ end of each native promoter (e.g., *BRN1p*) and 39 bp at 3′ end of each native promoter overlaps with 39 bp at 5′ end of each reporter gene, and 39 bp at 3′ of each reporter gene overlaps with 5′ end of the BamHI digested plasmid pRS41H. The primers sequences used to amplify the transcription factor binding sites, promoters, and reporter genes are given in Table S1a. In this study, the DNA assembler method was used for the in-vivo assembly of fragments of expression cassettes in *S. cerevisiae* [59]. This method requires the simple DNA preparation and one-step yeast transformation [59]. The workflow of the characterization of the strength of the promoters is given in Figure S1. The difference between the means was considered significant at p<0.05, and it was measured using the ANOVA test.

### 3.3. Characterization of Metal Ion Repressible Promoters

The metal ion repressible promoters *AFT1p* and *CTR1p* were characterized using reporter genes of *EGFP* and *LacZ*. The primers for metal ion repressible promoters and reporter genes were designed as that the 39 bp at 5′ end of each repressible promoter overlaps with 39 bp at 3′ end of BamHI digested pRS41H plasmid, whereas 39 bp at 3′ end of the repressible promoter overlaps with 39 bp at 5′ end of each reporter gene and 39 bp at 3′ of each reporter gene overlaps with 5′ end of BamHI digested pRS41H plasmid. Oligonucleotides sequences used for amplification of transcription factor binding sites, promoters, and reporter genes are given in Table S1b. In this study, the DNA assembler method was used for the in-vivo assembly of fragments of expression cassettes in *S. cerevisiae* [59]. This method requires the simple DNA preparation and one-step yeast transformation [59]. The workflow of the characterization of the strength of the promoters is given in Figure S1.

### 3.4. Characterization of the Strength of Engineered Promoters and Metal Ion Repressible Promoters

The strength of the engineered and metal ion repressible promoters was reckoned in terms of fluorescence intensity and relative mRNA level of *EGFP* as well as via enzymatic activity of β-galactosidase. Fluorescence intensity of the *EGFP* downstream of each promoter was reckoned using cytation-3 microplate reader (BioTek, Winooski, VT). For microplate reader analysis, single colony of each promoter harboring strain was inoculated in 10 mL test tube flask containing 5 mL YPD broth and incubated at 30 °C in shake rotary revolving at 170 rpm for overnight period, and then 1% inoculum of each strain was further grown in 20 mL Erlenmeyer flask containing 10 mL YPD broth and incubated at 30 °C in shake rotary revolution 170 rpm for 48 h. Afterward, 1 mL culture of each strain was harvested, then centrifuged at 1000g at 4 °C for 5 min and washed twice with ice-cold PBS buffer, and finally, the fluorescence intensity of *EGFP* was measured by using a microplate reader set at excitation wavelength 488 nm, and emission wavelength 509 nm. In addition to this, the strength of each promoter was also characterized by estimating the enzymatic activity of β-galactosidase, and for this purpose, the single colony of each promoter harboring strain was grown in 10 mL test tube containing 5 mL YPD broth and incubated at 30 °C in shake rotary revolving at 170 rpm for overnight period, and then 1% inoculum of each strain was further grown in 20 mL Erlenmeyer flask containing 10 mL YPD broth and incubated at 30 °C in shake rotary revolution 170 rpm for 48 h. After that, 1 mL culture of each strain was centrifuged at 12,000 rpm for 1 min, washed twice in 1 mL Z-buffer, and subsequently, the β-galactosidase activity was determined by using the Miller protocol.

The strength of *AFT1p* and *CTR1p* repressible promoters was also determined in terms of fluorescence intensity and relative mRNA level of *EGFP* and β-galactosidase activity. For this purpose, each strain was grown by using the above-mentioned growth protocols, and samples were also prepared by following the procedures above. However, in addition to this, the repressible promoters harboring strains were grown in SD histidine negative medium containing 200 uM FeSO_4_ and 200 uM CuSO_4_. *AFT1p* and *CTR1p* repressible promoters were mainly repressed by Fe^+2^ and Cu^+2^ ions, respectively, but were also weakly repressed by their alternate metal ions.

### 3.5. Yeast Engineering with Squalene and Ethanol Biosynthesis Pathways

Firstly, module-1 consisted of *HMG1* (hydroxymethylglutaryl CoA reductase), *IDI1* (isopentenyl diphosphate isomerase), *ERG20* (farnesyl diphosphate synthase) and *ERG9* (squalene synthase) vital genes of squalene biosynthesis pathway downstream of *TFBS_TEF-_HHF2p, TFBS_TEF-_IRA1p, TFBS_TEF-_RHO1p,* and *TFBS_TEF-_PET9p* promoters respectively, was integrated into the genome of *S. cerevisiae* using delta site sequences (Figure 1). Secondly, module-2 comprised of *PDC5* (pyruvate decarboxylase 5)*, ADH1* (alcohol dehydrogenase 1), *ADH4* (alcohol dehydrogenase 4), *ADH5* (alcohol dehydrogenase 5) and *ADH2* (alcohol dehydrogenase 2) vital genes of ethanol production pathway downstream of *TFBS_HHF2-_HHF2p, TFBS_HHF2-_IRA1p, TFBS_HHF2-_RHO1p, TFBS_HHF2-_PET9p* promoters respectively, was integrated into the genome of *S. cerevisiae* using delta site sequences (Figure 1).

The 300 ng of each expression cassette was used for the integration of module-1 and module-2 in the genomic DNA of *S. cerevisiae* (Figure 1). However, the module-1 was integrated into the genomic DNA of *S. cerevisiae* via delta sites, and they were amplified from chromosome 10 of *S. cerevisiae*. The expression cassettes of module-2 were also integrated at the delta site in the genome of yeast. The engineered strain containing module-1 and module-2 was selected on the YPD agar plates containing hygromycin and geneticin (G418), respectively. In this study, the DNA assembler method was used for the in-vivo assembly of fragments of expression cassettes in *S. cerevisiae* [59]. This method requires the simple DNA preparation and one-step yeast transformation [59].

The primers for construction of expression cassettes of each module were designed as that the 39 bp at 5′ end of the first expression cassette overlaps with 39 bp at 3′ end of the delta site whereas 39 bp at 3′ end of the first expression cassette overlaps with 39 bp at 5′ end of the second expression cassette and so on. The primers used for amplification of delta sites, functional genes, and promoters are given in Table S2.

### 3.6. Overexpression of the DGA1 in Engineered Strain

*DGA1* was overexpressed to enhance the squalene accumulation capacity of engineered strain. The primers used for the amplification of DNA fragments of the *DGA1* module were designed according to the procedure mentioned in Section 2.6. The design and primers of the *DGA1* module are given in Figure S2 and Table S5, respectively.

### 3.7. Downregulation of ERG1 Using Metal Ion Repressible Promoters

The metal ion repressible promoters such as *AFT1p* and *CTR1p* were employed to replace the native promoter of the *ERG1* gene (squalene monooxygenase) and downregulate its expression to optimize the production of squalene in FSE strain. The native promoter of the *ERG1* gene was replaced with *AFT1p* and *CTR1p* promoters through the homologous recombination pathway responsible for DSB in yeast [60,61]. The design of the modules used to replace *ERG1p* with *AFT1p* and *CTR1* is given in Figure S3. Primers used to amplify the DNA fragments of both modules are given in Table S4. The repression potential of each metal ion repressible promoter was measured at different metal ion concentrations such as 100, 150, 200, 250, and 300 uM of FeSO_4_ and CuSO_4_ in YPD media. All experiments were performed in biological triplicate.

### 3.8. Total RNA Extraction and qRT-PCR Analysis

For extraction of total RNA, a single colony of engineered strain FSE and promoters harboring strains were separately inoculated in 100 mL Erlenmeyer flask containing 25 mL YPD broth and incubated at 30 °C in shake rotary revolving at 170 rpm for an overnight period. Subsequently, 1% culture of the overnight grown strains was separately inoculated in 100 mL Erlenmeyer flask containing 25 mL YPD broth and incubated at 30 °C in shake rotary revolving at 170 rpm for 48 h. Yeast RNA Extraction Kit (OMEGA, Doraville, GA) was used for the extraction of total RNA from each sample. DNA contamination from RNA samples was excluded by adding 90 μL DNase I incubation buffer plus 10 μL DNase I into the RNA containing reaction tubes, afterward the reaction tubes were stored at 15 °C for 15 min.

The concentration of total RNA in each sample was reckoned with Nanodrop ND-1000 spectrophotometer (Thermo Scientific, Waltham, MA, USA), and the cDNA template was synthesized from one thousand nano-gram of total RNA using Transcriptor First Strand cDNA Synthesis Kit (Roche, Basel, Switzerland). Real-time qPCR reactions were performed on the LightCycler 480 real-time System (Roche, Basel, Switzerland), and reaction conditions were set as recommended by the SYBR *Premix Ex Taq*^TM^ manual (Takara Biomedical Technology Co., Ltd. Beijing, China). Relative quant of each gene was determined using housekeeping gene *ACT1* as a reference gene, and data were analyzed with Light Cycler Software (v.1.5). All assays were performed in triplicate, and reactions without reverse transcriptase were used as a negative control. The primers for qRT-PCR analysis were designed using Beacon Designer™ Free Edition software (PREMIER Biosoft, San Francisco, CA, USA), and their sequences are tabulated in Table S3.

### 3.9. Nile Red Staining of Lipid Droplets in Engineered Strain

Nile red staining is a reliable method to monitor the neutral lipid contents of living microorganisms such as yeast and microalgae. The enhanced biogenesis of lipid droplets due to the overexpression of *DGA1* and subsequent accumulation of squalene in engineered strain was monitored by staining them with Nile red dye. The protocol of staining of lipid droplets with Nile red staining was used as given elsewhere in the literature [62].

### 3.10. Extraction, Quantification and GC-MS Analysis of Squalene and Fusel Alcohols

For extraction of squalene and fusel alcohols single colony of FSE, FSE1 and WS strains were separately inoculated in a 10 mL test tube containing 5 mL YPD broth and incubated at 30 °C in shake rotary revolving at 170 rpm for an overnight period. Afterward, 1% culture of the overnight grown strains was separately inoculated in 50 mL Erlenmeyer flask containing 20 mL YPD broth and incubated at 30 °C in shake rotary revolving at 170 rpm for 48 h. Later, for extraction of squalene 5 mL cell culture of FSE, FSE1 and WS strains were centrifuged at 900 g for 1 min, and supernatant of FSE1 strain and WS was used for extraction of fusel alcohol including, 2-Phenylethanol, tyrosol, tryptophol, discarded. Afterward, 10 mL chloroform and 20 ul zirconium oxide beads (0.5 mm) were added in the cell pellets of FSE, FSE1, and WS strains, and subsequently, each cell pellet was lyzed using bullet blender, (Next Advance, Inc. Troy, NY, USA). The lyzed cells were centrifuged at 3500 g for 1 min, and the supernatant was concentrated up to 1 mL through evaporating rotary.

The production of squalene and fusel alcohol, e.g., 2-phenylethanol, tyrosol, tryptophol in FSE, and WS strains was determined using GCMS-ISQ trace 1300 (Thermo Fisher Scientific, Waltham, MA, USA) and TG-5MS column (Thermo Fisher Scientific, Waltham, MA, USA). The GC-MS was operated by programing it as, after injection of sample, the oven temperature was maintained at 80 °C for 1 min, then ramped to 280 °C at the rate of 20 °C /min and held at 280 °C for 15 min. Afterward, the temperature was further ramped to 300 °C at a rate of 20° C/min and sustained at 300 °C for 15 min. The peak of squalene and fusel alcohol, e.g., 2-phenylethanol, tyrosol, tryptophol in each sample were identified by comparing the GC-MS spectra of each sample with the authentic standard of squalene and fusel alcohol, e.g., 2-phenylethanol, tyrosol, tryptophol (Sigma-Aldrich). Furthermore, the fragmentation pattern of squalene and fusel alcohol, e.g., 2-phenylethanol, tyrosol, tryptophol in each sample, was also matched with fragmentation pattern of squalene and fusel alcohol, e.g., 2-phenylethanol, tyrosol, tryptophol in NIST library database. The production of squalene and fusel alcohol, e.g., 2-phenylethanol, tyrosol, tryptophol in FSE, and WS strains, was reckoned by constructing the standard curves of authentic standards of compounds above. All experiments were performed in triplicate.

### 3.11. Quantification of Ethanol

The production of ethanol in FSE, FSE1, and WS strains was determined by separately growing the single colony of each strain in 10 mL Erlenmeyer flask containing 5 mL YPD broth and incubated at 30 °C in shake rotary revolving at 170 rpm for an overnight period. Subsequently, 1% inoculum of each strain (OD_600_ 0.5) was separately inoculated in 100 mL Erlenmeyer flask containing 40 mL YPD broth and fermented at 30 °C in shake rotary revolving at 170 rpm. The 100 uL sample of each strain was collected after an interval of 3 h, 6 h, 12 h, 24 h, 36 h, 48 h, 60 h, 72 h, and 80 h. Later, each sample was centrifuged at 5000 g for 1 min, and the production of ethanol in FSE, FSE1, and WS strains was measured using SBA-40E biosensor (Institute of Biology, Shandong Academy of Sciences, China). All experiments were performed in biological triplicate.

## 4. Conclusions

Findings of this study display that ligation of transcription factor binding sites (TFBS) from strong promoters upstream of yeast promoters increased the strength of native promoters. Engineered promoters were employed to co-overexpress the squalene and ethanol production pathways in *S. cerevisiae*. Employment of metal ion repressible promoters downregulated the expression of *ERG1* and enhanced the production of squalene up to 255.11 mg/L in FSE strain. Co-overexpression of squalene and ethanol pathways enabled the co-production of squalene and ethanol up to 255.11 mg/L and 40.2 mg/L, respectively, in FSE1 strain. Moreover, FSE1 strain synergistically downregulated the expression of the fusel alcohol pathway, perhaps to divert the metabolic flux toward engineered pathways. It is assumed that FSE1 strain downregulated the expression of the fusel alcohol pathway by downregulating the expression of *GCN4.*

Our prospective study will focus on the identification and characterization of vital components of regulatory networks associated with *GCN4* based regulation of fusel alcohol pathway.

## Supplementary Materials

The following are available online at https://www.mdpi.com/2218-1989/10/2/56/s1, Figure S1: Work flow of characterization of strength of engineered and repressible promoters; Figure S2: Design of the module used to overexpress the *DGA1* in engineered strain FSE; Figure S3: Design of the module used to swipe the ERG1 promoter with AFT1p and CTR1p in engineered strain FSE; Figure S4: Nile red staining dye was used to visualize the lipid droplets biogenesis and accumulation of squalene in engineered strain FSE compared with wild strain; Figure S5: (a) Confocal microscopic analysis of wild type yeast promoters. (b) Confocal microscopic analysis of engineered promoters containing TFBS of HHF2p; Figure S6: GCMS analysis of metabolites; Table S1a: List of the primers designed to amplify and construct the engineered promoters; Table S1b: List of the primers designed to amplify the metal ions repressible promoters; Table S2: List of primer designed to amplify the genes of module-1; Table S3: List of primer designed for qRT-PCR of overexpressed genes of the squalene biosynthesis pathway, ethanol production pathway and fusel alcohol pathway; Table S4: List of primer designed to amplify the DNA fragments for modules given in supplementary Figure S1; Table S5: List of primer designed to amplify the DNA fragments of DGA1 module; Table S6: Fluorescence intensity of wild type promoters and engineered promoters.
