# Supplementary material for: Self-Redirection of Metabolic Flux toward Squalene and Ethanol Pathways by Engineered Yeast
Source: Metabolites. 2020 Feb 1;10(2):56. doi: 10.3390/metabo10020056 (PMC7074498; doi:10.3390/metabo10020056)
Supplement: Supplementary file 1 [file metabolites-10-00056-s001.pdf]

Self-Re-direction of Metabolic Flux Toward Squalene and Ethanol Pathways by Engineered Yeast

Robina Manzoor[1], Maqbool Ahmed[4], Ullah Kaleem[1,5], Imdad Kaleem[6], Aamir Rasool[1,2,3]*

[1]School of Life Science, Beijing Institute of Technology, Beijing 100081, PR China

[2] Institute for Synthetic Biosystem, School of Chemistry and Chemical Engineering, Beijing Institute of Technology, Beijing 100081, PR China

[3]Institute of Biochemistry, University of Balochistan, Quetta 87300, Pakistan

[4]Department of Tuberculosis, Bolan University of Medical and Health Sciences, Quetta 87300, Pakistan

[5]Department of Microbiology, University of Balochistan, Quetta 87300, Pakistan

[6]Department of Bioscience, COMSATS Institute of Information Technology (CIIT), Islamabad 45550, Pakistan

Supplementary Material

Supplementary table.S1a. List of the primers designed to amplify and construct the engineered promoters.

HHF2t (-300 to -669bp)

HHF2t-F: GTATCGATAAGCTTGATATCGAATTCCTGCAGCCCGGGGGAAC GGACAACTGTGGTCGT
HHF2t-R: TATTTTATTGTATTGATTG
LAC Z-F: ATGACCATGATTACGGATTC
LAC Z-R: TGGAGCTCCACCGCGGTGGCGGCCGCTCTAGAACTAGTG TTATTTTTGACACCAGACCA
TFBS$_{HHF2}$-COQ1-F: AAAAGAGTAGCAAAAACAACAATCAATACAATAAAATA ACCGTCTTTTACCTGTGTTT
TFBS$_{HHF2}$-COQ1-R: TTGTAAAACGACGGCCAGTGAATCCGTAATCATGGTCAT CGTCCCACTGTACTCAATTC
TFBS$_{HHF2}$-IRA1-F: AAAAGAGTAGCAAAAACAACAATCAATACAATAAAATA GCGAGCCGACACCGCCTGGA
TFBS$_{HHF2}$-IRA1-R: TTGTAAAACGACGGCCAGTGAATCCGTAATCATGGTCAT TTTAGAAAGAGCCTTAATCT
TFBS$_{HHF2}$-MAK5-F: AAAAGAGTAGCAAAAACAACAATCAATACAATAAAATA TCCGCTGTATTAGTGGTATC
TFBS$_{HHF2}$-MAK5-R: TTGTAAAACGACGGCCAGTGAATCCGTAATCATGGTCAT GATGGAAAAACTGATTATG
TFBS$_{HHF2}$-RER-F: AAAAGAGTAGCAAAAACAACAATCAATACAATAAAATA GAATTGAGTACAGTGGGACG
TFBS$_{HHF2}$-RER-R: TTGTAAAACGACGGCCAGTGAATCCGTAATCATGGTCAT ACCGTCTTTTACCTGTGTTT
TFBS$_{HHF2}$-RHO1-F: AAAAGAGTAGCAAAAACAACAATCAATACAATAAAATA TTCCCTCATTTCCAATAACA
TFBS$_{HHF2}$-RHO1-R: TTGTAAAACGACGGCCAGTGAATCCGTAATCATGGTCAT CTTTCTAGTATAATTTTTAA
TFBS$_{HHF2}$-PET9-F: AAAAGAGTAGCAAAAACAACAATCAATACAATAAAATA TAACGAAATAATTTCCTTTT
TFBS$_{HHF2}$-PET9-R: TTGTAAAACGACGGCCAGTGAATCCGTAATCATGG GGCTATTTGCTTATATGTAT
TFBS$_{HHF2}$-USA3-F: AAAAGAGTAGCAAAAACAACAATCAATACAATAAAATA TTTCCGTGTGATGGGCCTTC
TFBS$_{HHF2}$-USA3-R: TTGTAAAACGACGGCCAGTGAATCCGTAATCATGG TTTCACAGTACAAATATTTC
TFBS$_{HHF2}$-ATP16-F: AAAAGAGTAGCAAAAACAACAATCAATACAATAAAATA TACTTTCAGACGGGCAGTTT
TFBS$_{HHF2}$-ATP16-R: TTGTAAAACGACGGCCAGTGAATCCGTAATCATGG TTTCCTATCCTCACGGCTA
TFBS$_{HHF2}$-CMD1-F: AAAAGAGTAGCAAAAACAACAATCAATACAATAAAATA ATGTCCTAATGAATTCAAGC
TFBS$_{HHF2}$-CMD1-R: TTGTAAAACGACGGCCAGTGAATCCGTAATCATGG TGTACTTTTTTATTTGTATT
TFBS$_{HHF2}$-RIM2-F: AAAAGAGTAGCAAAAACAACAATCAATACAATAAAATA TAAAAAATAAATTGTGTCTA
TFBS$_{HHF2}$-RIM2-R: TTGTAAAACGACGGCCAGTGAATCCGTAATCATGG CTTTATCTCCTTGAAAAATT
TFBS$_{HHF2}$-BRN-F: AAAAGAGTAGCAAAAACAACAATCAATACAATAAAATA ATAGTGTTTGCTCCCCTTAC
TFBS$_{HHF2}$-BRN-R: TTGTAAAACGACGGCCAGTGAATCCGTAATCATGG TCTTTTAATCTTAACCGATT
TFBS$_{HHF2}$-HHF2-F: AAAAGAGTAGCAAAAACAACAATCAATACAATAAAATA TGTTTGCTTGGATCCTTTAG
TFBS$_{HHF2}$-HHF2-R: TTGTAAAACGACGGCCAGTGAATCCGTAATCATGG TATTTTATTGTATTGATTGT
TFBS $_{HHF2}$-GRS1: F- AAAAGAGTAGCAAAAACAACAATCAATACAATAAAATA ATAGTGTTTG CTCCCCTTAC

TFBS $_{HHF2}$-GRS1-R: TTGTAAAACGACGGCCAGTGAATCCGTAATCATGG TCTTTTAATCTTAACCGATT

TEF1t (-300 to -579bp)

TEF1t-F: GTATCGA TAAGCTTGAT ATCGAATTCC TGCAGCCCGG ACAATGCATA CTTTGTACGT
TEF1t-F: TTTGTAATTAAAACTTAGAT
LAC Z-F: ATGACCATGATTACGGATTC
LAC Z-R: TGGAGCTCCACCGCGGTGGCGGCCGCTCTAGAACTAGTG TTATTTTTGACACCAGACCA
TFBS$_{TEF1}$-COQ1-F: AAGAAAGCAT AGCAATCTA A TCTAAGTTTT AATTACAAA ACCGTCTTTTACCTGTGTTT
TFBS$_{TEF1}$-COQ1-R: TTGTAAAACG ACGGCCAGTG AATCCGTAAT CATGGTCAT CGTCCCACTGTACTCAATTC
TFBS$_{TEF1}$-IRA1-F: AAGAAAGCAT AGCAATCTA A TCTAAGTTTT AATTACAAA GCGAGCCGAC ACCGCCTGGA
TFBS$_{TEF1}$-IRA1-R: TTGTAAAACG ACGGCCAGTG AATCCGTAAT CATGGTCAT TTTAGAAAGAGCCTTAATCT
TFBS$_{TEF1}$-MAK5-F: AAGAAAGCAT AGCAATCTA A TCTAAGTTTT AATTACAAA TCCGCTGTAT TAGTGGTATC
TFBS$_{TEF1}$-MAK5-R: TTGTAAAACG ACGGCCAGTG AATCCGTAAT CATGGTCAT GATGGAAAAACTGATTATG
TFBS$_{TEF1}$-RER-F: AAGAAAGCAT AGCAATCTA A TCTAAGTTTT AATTACAAA GAATTGAGTACAGTGGGACG
TFBS $_{TEF1}$-RER-R: TTGTAAAACG ACGGCCAGTG AATCCGTAAT CATGGTCAT ACCGTCTTTTACCTGTGTTT
TFBS $_{TEF1}$-RHO1-F: AAGAAAGCAT AGCAATCTA A TCTAAGTTTT AATTACAAA TTCCCTCATT TCCAATAACA
TFBS $_{TEF1}$-RHO1-R: TTGTAAAACG ACGGCCAGTG AATCCGTAAT CATGGTCAT CTTTCTAGTATAATTTTTAA
TFBS $_{TEF1}$-PET9-F: AAGAAAGCAT AGCAATCTA A TCTAAGTTTT AATTACAAA TAACGAAATA ATTTCCTTTT
TFBS $_{TEF1}$-PET9-R: TTGTAAAACG ACGGCCAGTG AATCCGTAAT CATGGTCAT GGCTATTTGCTTATATGTAT
TFBS $_{TEF1}$-USA3-F: AAGAAAGCAT AGCAATCTA A TCTAAGTTTT AATTACAAA TTTCCGTGTG ATGGGCCTTC
TFBS $_{TEF1}$-USA3-R: TTGTAAAACG ACGGCCAGTG AATCCGTAAT CATGGTCAT TTTCACAGTACAAATATTTC
TFBS $_{TEF1}$-ATP16-F: AAGAAAGCAT AGCAATCTA A TCTAAGTTTT AATTACAAA TACTTTCAGA CGGGCAGTTT
TFBS $_{TEF1}$-ATP16-R: TTGTAAAACG ACGGCCAGTG AATCCGTAAT CATGGTCAT TTTCCTATCCTCACGGCTA
TFBS $_{TEF1}$-CMD1-F: AAGAAAGCAT AGCAATCTA A TCTAAGTTTT AATTACAAA ATGTCCTAAT GAATTCAAGC
TFBS $_{TEF1}$-CMD1-R: TTGTAAAACG ACGGCCAGTG AATCCGTAAT CATGGTCAT TGTACTTTTTTATTTGTATT
TFBS $_{TEF1}$-RIM2-F: AAGAAAGCAT AGCAATCTA A TCTAAGTTTT AATTACAAA TAAAAAATAA ATTGTGTCTA
TFBS $_{TEF1}$-RIM2-R: TTGTAAAACG ACGGCCAGTG AATCCGTAAT CATGGTCAT CTTTATCTCCTTGAAAAATT
TFBS $_{TEF1}$-BRN-F: AAGAAAGCAT AGCAATCTA A TCTAAGTTTT AATTACAAA ATAGTGTTTG CTCCCCTTAC
TFBS $_{TEF1}$-BRN-R: TTGTAAAACG ACGGCCAGTG AATCCGTAAT CATGGTCAT TCTTTTAATCTTAACCGATT
TFBS $_{TEF1}$-HHF2-F: AAGAAAGCAT AGCAATCTA A TCTAAGTTTT AATTACAAA TGTTTGCTTG GATCCTTTAG
TFBS $_{TEF1}$-HHF2-R: TTGTAAAACG ACGGCCAGTG AATCCGTAAT CATGGTCAT TATTTTATTGTATTGATTGT
TFBS $_{TEF1}$-GRS1: FAAGAAAGCAT AGCAATCTA A TCTAAGTTTT AATTACAAA ATAGTGTTTG CTCCCCTTAC
TFBS $_{TEF1}$-GRS1-R: TTGTAAAACG ACGGCCAGTG AATCCGTAAT CATGGTCAT TCTTTTAATCTTAACCGATT

Supplementary table.S1b. List of the primers designed to amplify the metal ions repressible promoters.

Primers for amplification of AFT1p repressible promoters

AFT1-F: TATTACAACTTTTTTTACTTCTTGCTCATTAGAAAGAAAGATCACGTAAAACAACAACC
AFT1-R1: CACCACCCCGGTGAACAGCTCCTCGCCCTTGCTCACCATTGTCGTAGATTTTTCTGTTA
AFT1-R2: TTGTAATACGACGGCCAGTGAATCCGTAATCATAGTCATTGTCGTAGATTTTTCTGTTA
EGFP-F: ATTTTTAAAAACAAAAAAATAACAGAAAAATCTACGACAATGGTGAGCAAGGGCGAGGA
EGFP-R: TCCACCGCGGTGGCGGCCGCTCTAGAACTAGTGGATCCCTTACTTGTACAGCTCGTCCA
LacZ-F: ATTTTTAAAAACAAAAAAATAACAGAAAAATCTACGACAATGACTATGATTACGGATTC
LacZ-R: TCCACCGCGGTGGCGGCCGCTCTAGAACTAGTGGATCCCTTATTTTTGACACCAGACCA

Primers for amplification of CTR1p repressible promoters

CTR1-F: GTATCGATAAGCTTGATATCGAATTCCTGCAGCCCGGGGACCGTATTTTGCTCGTTCCA

```
CTR1-R1: CACCACCCCGGTGAACAGCTCCTCGCCCTTGCTCACCATTTTGAATGTCAAATATAATA
CTR1-R2: TTGTAATACGACGGCCAGTGAATCCGTAATCATAGTCATTTTGAATGTCAAATATAATA
 EGFP-F: TAGAAAATAAAAAAAAGTGTATTATATTTGACATTCAAAATGGTGAGCAAGGGCGAGGA
 EGFP-R: TCCACCGCGGTGGCGGCCGCTCTAGAACTAGTGGATCCCTTACTTGTACAGCTCGTCCA
 LacZ-F: TAGAAAATAAAAAAAAGTGTATTATATTTGACATTCAAAATGACTATGATTACGGATTC
 LacZ-R: TCCACCGCGGTGGCGGCCGCTCTAGAACTAGTGGATCCCTTATTTTTGACACCAGACCA
```

Note: R1 and R2 primers were designed to overlap with EGFP and LacZ reporter genes respectively.

Supplementary table.S2. List of primer designed to amplify the genes of module-1.

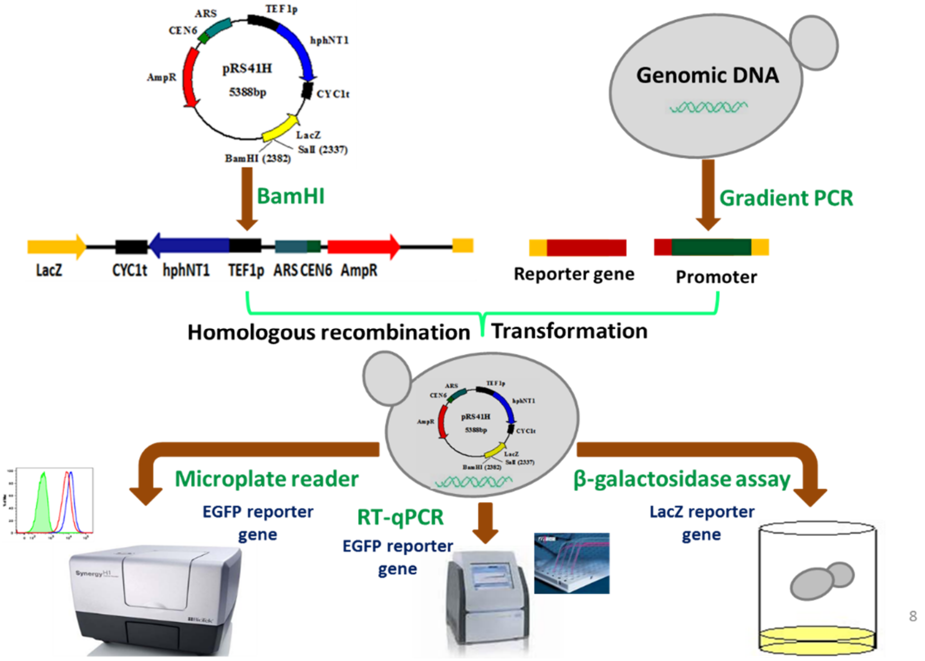

Supplementary figure.S1. Work flow of characterization of strength of engineered and repressible promoters.

Construction of Module-1 for engineering of FSE strain

Delta-F: GACTATTCCTCATTCCGTTT
Delta-R: AGACTGTCAAGGAGGGTATTCTGGGCCTCCATGTCGCTGTTAGTAGTATACTATCACAT
HbH-F: TGTTATCTTTTTACACCACATGTGATAGTATACTACTAACAGCGACATGGAGGCCCAGA
HbH-R: TATTTTATTGTATTGATTGTTGTTTTTGCTACTCTTTTCGTCCCAAAACCTTCTCAAG
TFBS$_{TEF1}$-HHF2-F: AAAAGAGTAGCAAAAACAACAATCAATACAATAAAATA TGTTTGCTTGGATCCTTTAG
TFBS-$_{TEF1}$-HHF2-R: TTGTAAAACGACGGCCAGTGAATCCGTAATCATGG TATTTTATTGTATTGATTGT
HMG1-F: CCATGATTACGGATTCACTGGCCGTCGTTTTACAA ATGACTGCTCCAAATAAAGA
HMG1-R: ATAAATTGAAGCTTGGTCGTTCCGACCAATACCGTAGGGTTAGGATTTAATGCAGGTGACG
ERG19t-F: CGTTTGAAAGATGGGTCCGTCACCTGCATTAAATCCTAACCCTACGGTATTGGTCGGAA
ERG19t-R: TATTTTATTGTATTGATTGTTGTTTTTGCTACTCTTTTTGGCTGCTGAGATCTTTAAG
TFBS-$_{TEF1}$IRA1-F: AAAAGAGTAGCAAAAACAACAATCAATACAATAAAATAGCGAGCCGACACCGCCTGGA
TFBS-$_{TEF1}$-IRA1-R: TTGTAAAACGACGGCCAGTGAATCCGTAATCATGGTCATTTTAGAAAGAGCCTTAATCT
IDI1-F: ATGACCATGATTACGGATTCACTGGCCGTCGTTTTACAAATGACTGCCGACAACAATAG
IDI1-R: CTTCTTTTTCAAGAGAATACCAATGACGTATGACTAAGTTTATAGCATTCTATGAATTT
HMG1t-F: TTGACCATGAGAAAATATACAGAATGTGAACTTAGTCATACGTCATTGG
HMG1t-R: GGAAGATTTTCACTCATAAGGGAAAGGAACCGCATGACTGGAAGATTTTCACTCATAAG

TFBS-$_{TEF1}$-RHO1-F: AAAAGAGTAGCAAAAACAACAATCAATACAATAAAATATTCCCTCATTTCCAATAACA
TFBS-$_{TEF1}$-RHO1-R: TTGTAAAACGACGGCCAGTGAATCCGTAATCATGGTCATCTTTCTAGTATAATTTTTAA
ERG20-F: ATGACCATGATTACGGATTCACTGGCCGTCGTTTTACAAATGGCTTCAGAAAAAGAAAT
ERG20-R: TTACAAGAGAAGCAAATAGTAAATGTATATTCTTGTAAATATTATAACATATATACGA
MRH4t-F: ACTGCGTTCTTGAACAAAGTTTACAAGAGAAGCAAATAGTAAATGTATATTCTTGTAAA
MRH4t-R: TATTTTATTGTATTGATTGTTGTTTTTGCTACTCTTTTAAGCAATCGTTCTTTTATCC
TFBS-$_{TEF1}$-PET9-F: AAAAGAGTAGCAAAAACAACAATCAATACAATAAAATATAACGAAATAATTTCCTTTT
TFBS-$_{TEF1}$-PET9-R: TTGTAAAACGACGGCCAGTGAATCCGTAATCATGGGGCTATTTGCTTATATGTAT
ERG9-F: CCATGATTACGGATTCACTGGCCGTCGTTTTACAA ATGGGAAAGCTATTACAATT
ERG9-R: CTTTAATGTTCTTTAGGTATATATTTAAGAGCGATTTGTTCACGCTCTGTGTAAAGTGT
PGI1t-F: CTTGGGTTTTATTATATATACACTTTACACAGAGCGTGAACAAATCGCCTTAAATATA
PGI1t-R: GGAATCCCAACAATTATCTCAAAACTCACTTATATCTCATTTTAAACAGTTGATGAGAA
Delta1-2-F: CTTGAACTTGCGAAAAAGGTTCTCATCAACTGTTTAAAATGAGATATAAGTGAGTTTTG
Delta1-2-R: GTATTATTAGTATGTATAGA

Construction of Module-2 for engineering of FSE strain

Delta6-1-F: TGTTAATATTCATTGATCCT
Delta6-1-R: AGACTGTCAAGGAGGGTATTCTGGGCCTCCATGTCGCTGTGTTGGAATAAAAATCAACT
KamMX-F: ATACTAGTTAGTAGATGATAGTTGATTTTTATTCCAACACAGCGACATGGAGGCCCAGA
KamMX-R: TATTTTATTGTATTGATTGTTGTTTTTGCTACTCTTTTTTTCGACACTGGATGGCGGC
TFBS$_{HHF2}$-HHF2-F: AAAAGAGTAGCAAAAACAACAATCAATACAATAAAATA TGTTTGCTTGGATCCTTTAG
TFBS$_{HHF2}$-HHF2-R: TTGTAAAACGACGGCCAGTGAATCCGTAATCATGG TATTTTATTGTATTGATTGT
PDC5-F: CTGTCGATTCGATACTAACGCCGCCATCCAGTGTCGAAA ATGTCTGAAA TAACCTTAGG
PDC5-R: CTACTAACGCTAAACAATAACCCTACGGTATTGGTCGGAA CGACCAAGCTTCAATTTAT
ERG19t-F: AGCTCAATTGACTGCCGCTACTAACGCTAAACAATAA CCCTACGGTA TTGGTCGGAA
ERG19t-R: TATTTTATTGTATTGATTGTTGTTTTTGCTACTCTTTTTGGCTGCTGAGATCTTTAAG
TFBS$_{HHF2}$-IRA1-F: AAAAGAGTAGCAAAAACAACAATCAATACAATAAAATA GCGAGCCGACACCGCCTGGA
TFBS$_{HHF2}$-IRA1-R: TTGTAAAACGACGGCCAGTGAATCCGTAATCATGGTCAT TTTAGAAAGAGCCTTAATCT
ADH1-F: ATGACCATGATTACGGATTCACTGGCCGTCGTTTTACAA ATGTCTATCC CAGAAACTCA
ADH1-R: CTTCTTTTTCAAGAGAATACCAATGACGTATGACTAAGTTTATTTAGAAGTGTCAACAA
HMG1t-F: TCAAATCGTTGGTAGATACGTTGTTGACACTTCTAAATAA ACTTAGTCAT ACGTCATTGG
HMG1t-R: TATTTTATTGTATTGATTGTTGTTTTTGCTACTCTTTTGGAAGATTTTCACTCATAAG
TFBS$_{HHF2}$-RHO1-F: AAAAGAGTAGCAAAAACAACAATCAATACAATAAAATA TTCCCTCATTTCCAATAACA
TFBS$_{HHF2}$-RHO1-R: TTGTAAAACGACGGCCAGTGAATCCGTAATCATGGTCAT CTTTCTAGTATAATTTTTAA
ADH4-F: ATGACCATGATTACGGATTCACTGGCCGTCGTTTTACAA ATGTCTTCCG TTACTGGGTT
ADH4-R: TCGTATATATGTTATAATATTTACAAGAATATACATTTATTAATATTCATAGGCTTTCT
MRH4-F: CAAGTGGTTG CCATTATCAA GAAAGCCTAT GAATATTAA TAAATGTATA TTCTTGTAAA
MRH4-R: TATTTTATTGTATTGATTGTTGTTTTTGCTACTCTTTTAAGCAATCGTTCTTTTATCC
TFBS$_{HHF2}$-PET9-F: AAAAGAGTAGCAAAAACAACAATCAATACAATAAAATA TAACGAAATAATTTCCTTTT
TFBS$_{HHF2}$-PET9-R: TTGTAAAACGACGGCCAGTGAATCCGTAATCATGG GGCTATTTGCTTATATGTAT
ADH2-F: TTGTAAAACGACGGCCAGTGAATCCGTAATCATGG ATGTCTATTC CAGAAACTCA
ADH2-R: CTTTAATGTTCTTTAGGTATATATTTAAGAGCGATTTGTTTATTTAGAAGTGTCAACAA
PGI1t-F: CCA AATTGCTGGT AGATACGTTG TTGACACTTC TAAATAAACAAATCGCTCTTAAATATA
PGI1t-R: GGAATCCCAACAATTACATCAAAATCCACATTCTCTTCATTTTAAACAGTTGATGAGAA
Delta6-2-F: CTTGAACTTGCGAAAAAGGTTCTCATCAACTGTTTAAAATGAAGAGAAT GTGGATTTTG
Delta6-2-R: TATAAAATGATGATAATAAT

Supplementary table.S3. List of primer designed for qRT-PCR of overexpressed genes of the squalene biosynthesis pathway, ethanol production pathway and fusel alcohol pathway.

HMG1-F: TTTGCCCGTTGGTGTTATAG
HMG1-R: CATTGATTGCCTTACAGCCA
IDI1-F: ATCGTGCATTCTCCGTCTTTA
IDI1-R: ACCCTTCAAACCTAATTCGTC

ERG20-F: TTAATCACTGCACCTGAAGAC
ERG20-R: CGACAGGCAAGTAGAAAGAAT
ERG9-F: GTGAAGCCAAATGAAACTCCA
ERG9-R: AAACCCAAGAAGAACGGACA
PDC5-F: GAACAACACCCAAGATACGG
PDC5-R: GAAATCAGACAACAAAGCACC
ADH1-F: GCTTCTACCAGATACGTTAGAG
ADH1-R: CAACAATAGAGATGGACTTGAC
ADH4-F: GACTAACCCAGTTCAATTCACC
ADH4-R: ATAAGGCACACGCATAATTGAC
ADH2-F: AAGGCTTTGAAGTCTGCCAA
ADH2-R: TCCTTCTCTTTGGTGAAGTC

ARO8-F AAATACAACTCAGACCCTTACCAG
ARO8-R AATCTCTTCAGACCTTCAGTCAG
ARO9-F TTTCCTTCCTATAATCCGCAAGAG
ARO9-R GTAGTAATGTCGGCCAAATCCA
ARO10-F TGGTGATGTTGTCGTTTGTG
ARO10-R ATTGATGTGAGCGTTTGAGTG
BAT1-F ACTAGAAACCCAAATCCATCCA
BAT1-R GGCAGACTTGTTCATACGGG
BAT2-F GAAGCCACTGATTATGCCAC
BAT2-R GAATCCCTAGTAACACCTTCCA
GCN4-F: CCACTTCTACTGCCAAACCA
GCN4-R: GAAAGATTCCACTACAGCGTC

Supplementary table.S4. List of primer designed to amplify the DNA fragments for modules given in supplementary figure-S1.

Module to swap the AFT1p with ERG1p

ERG1-UP-F: ATGTCTGCTGTTAACGTTGC
ERG1-UP-R: AATCATTACCGAGGCATAAAAAAATATAGAGTGTACTAGAATGTTTCTCAAGTTGTTCA
HIS3-F: TTTGTTCATGGTAGATTCTTGAACAACTTGAGAAACATTCTAGTACACTCTATATTTTT
HIS3-R: TGCAGTTCAACCTCACTCGCGTGCGGCGGTGTGAGGTGCCCTGATGCGGTATTTTCTCC
AFT1-F: ATACCGCACAGATGCGTAAGGAGAAAATACCGCATCAGGGCACCTCACACCGCCGCACG
AFT1-R: GGCATTAATCAATTCAGGTGCAACGTTAACAGCAGACATTGTCGTAGATTTTTCTGTTA
ERG1-F: ATTTTTAAAAACAAAAAAATAACAGAAAAATCTACGACAATGTCTGCTGTTAACGTTGC
ERG1-R: CAAAGTACACGTTAAAATTCAGCTCGGGTATGTTAAGTATTAACCAATCAACTCACCAA
YRA1t-F: GTATTCACCCCATTTTTGTTTGGTGAGTTGATTGGTTAATACTTAACATACCCGAGCTG
YRA1t-R: GACAGTCATACCACCACCAGTCAATGGATGTCTCATATTTACCATTTGCAAGACATTGT
ERG1-Dn-F: CTTTCAACAAAAATATTATACAATGTCTTGCAAATGGTAAATATGAGACATCCATTGAC
ERG1-Dn-R: TTAACCAATCAACTCACCAA

Module to swap the CTR1p with ERG1p

ERG1-UP-F: ATGTCTGCTGTTAACGTTGC
ERG1-UP-R: AATCATTACCGAGGCATAAAAAAATATAGAGTGTACTAGAATGTTTCTCAAGTTGTTCA
HIS3-F: TTTGTTCATGGTAGATTCTTGAACAACTTGAGAAACATTCTAGTACACTCTATATTTTT
HIS3-R: AACAAAAACGTGGAAAGGCTGGAACGAGCAAAATACGGTCCTGATGCGGTATTTTCTCC
CTR1-F: ATACCGCACAGATGCGTAAGGAGAAAATACCGCATCAGG ACCGTATTTT GCTCGTTCCA
CTR1-R: GGCATTAATCAATTCAGGTGCAACGTTAACAGCAGACATTTTGAATGTCAAATATAATA
ERG1-F: TAGAAAATAAAAAAAAGTGTATTATATTTGACATTCAAAATGTCTGCTGTTAACGTTGC
ERG1-R: CAAAGTACACGTTAAAATTCAGCTCGGGTATGTTAAGTATTAACCAATCAACTCACCAA
YRA1t-F: GTATTCACCCCATTTTTGTTTGGTGAGTTGATTGGTTAATACTTAACATACCCGAGCTG
YRA1t-R: GACAGTCATACCACCACCAGTCAATGGATGTCTCATATTTACCATTTGCAAGACATTGT

ERG1-Dn-F: CTTTCAACAAAAATATTATACAATGTCTTGCAAATGGTAAATATGAGACATCCATTGAC
ERG1-Dn-R: TTAACCAATCAACTCACCAA

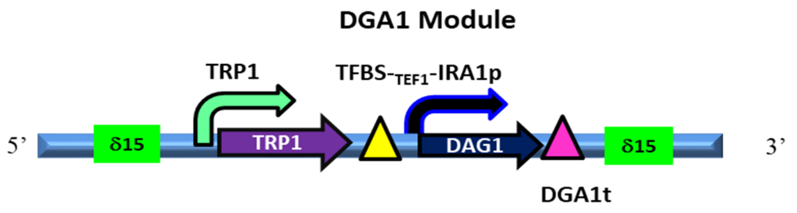

Supplementary figure.S2. Design of the module used to overexpress the *DGA1* in engineered strain FSE.

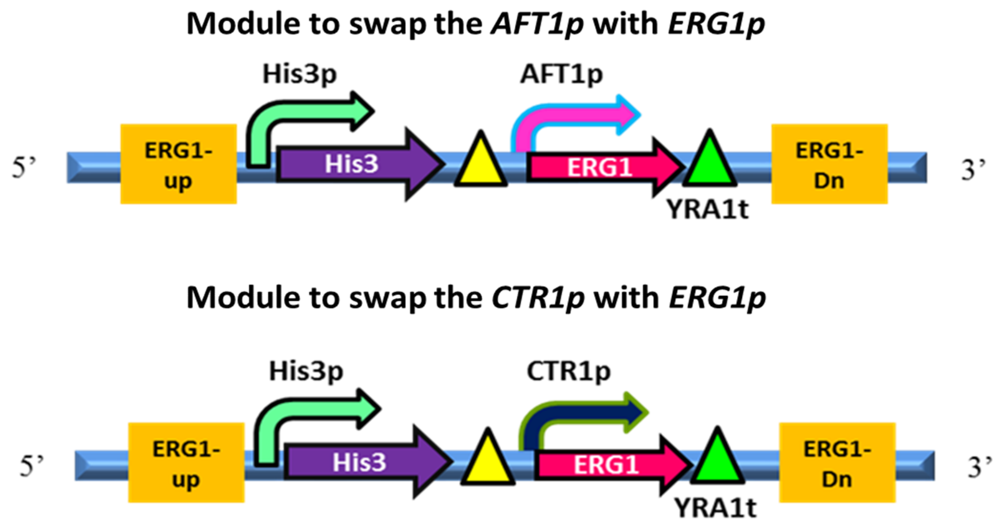

Supplementary figure.3. Design of the module used to swipe the ERG1 promoter with AFT1p and CTR1p in engineered strain FSE.

Supplementary table.S5. List of primer designed to amplify the DNA fragments of DGA1 module.

DELTA15-1-F: TATGTTTATATTCATTGATC
DELTA15-1-R: ATTAAATGCTTCCTATATTATATATATATAGTAATGTCGTTTGTTGGAATAAAAATCCACT
TRP1-F: CTATTAGTTGATAGACGATAGTGGATTTTTATTCCAACAAACGACATTACTATATATAT
TRP1-R: ACCACTTTCCCGGTGCCAAACGACCACAGTTGTCCGTTCCCTGATGCGGTATTTTCTCC
$TFBS-_{TEF1}IRA1$-F: ATACCGCACAGATGCGTAAGGAGAAAATACCGCATCAGGGGGAACGGACAACTGTGGTCGT
$TFBS-_{TEF1}$-IRA1-R: TTCTAGCCTTCCCATTACGTCAATGCTGACTGAATCTATTTTAGAAAGAGCCTTAATCT
DGA1-F: CAGCATATAACATACAACAAGATTAAGGCTCTTTCTAAAATAGATTCAGTCAGCATTGA
DGA1-R: GGAATCCCAACAATTATCTCAACATTCACATATTTCTCATTACCCAACTATCTTCAATTC
DELTA15-2-F: TATGGGGTACCGGATGCAGAATTGAAGATAGTTGGGTAATGAGAAATATGTGAATGTTG
DELTA15-2-R: TAAAACGGAATGAGGAATAA

Supplementary table.S6. Fluorescence intensity of wild type promoters and engineered promoters.

#	wild type promoters	Size of Wild type	Average Fluorescence Intensity	Engineered promoters with	Average Fluorescence Intensity	% increase in fluoresce	Engineered promoters with	Average Fluorescence Intensity	% increase in fluoresc

		promoters (bp)	(A.U)of wild type promoters	TFBS-TEF1 (-300 to -579 bp)	(A.U)of engineered promoters	nce intensity of engineered promoters	TFBS-HHF2	(A.U) of engineered promoters	ence intensity of engineered promoters
1	HHF2	669	402.2	HHF2-TEF1	662.39	56.39%	HHF2-HHF2	550.55	36.88%
2	IRA1	396	231.48	IRA1-TEF1	357.67	101.7%	IRA1-HHF2	369.65	59.69%
3	RHO1	441	177.2	RHO1-TEF1	343	95.51%	RHO1-HHF2	268.34	51.43%
4	PET9	490	140.31	PET9-TEF1	373.33	164.89%	PET9-HHF2	281.21	100.42%
5	CMD1	245	128.08	CMD1-TEF1	253.33	108.69%	CMD1-HHF2	246.58	92.52%
6	ATP16	719	118.81	ATP16-TEF1	231.67	97.63%	ATP16-HHF2	220.68	85.74%
7	USA3	385	110.95	USA3-TEF1	198.34	96.49%	USA3-HHF2	195.67	76.36%
8	RER2	240	100.99	RER2-TEF1	183.33	81.53%	RER2-HHF2	182.54	80.75%
9	COQ1	240	100.2	COQ1-TEF1	163.54	63.21%	COQ1-HHF2	148.83	48.53%
10	RIM1	428	98.01	RIM1-TEF1	122	24.48%	RIM1-HHF2	123.95	26.47%
11	GRS1	602	95.75	GRS1-TEF1	116.33	21.49%	GRS1-HHF2	118.62	23.89%
12	MAK5	278	87.37	MAK5-TEF1	106	21.32%	MAK5-HHF2	124.46	42.45%
13	BRN1	400	83.17	BRN1-TEF1	106	27.45%	BRN1-HHF2	125.23	50.57%

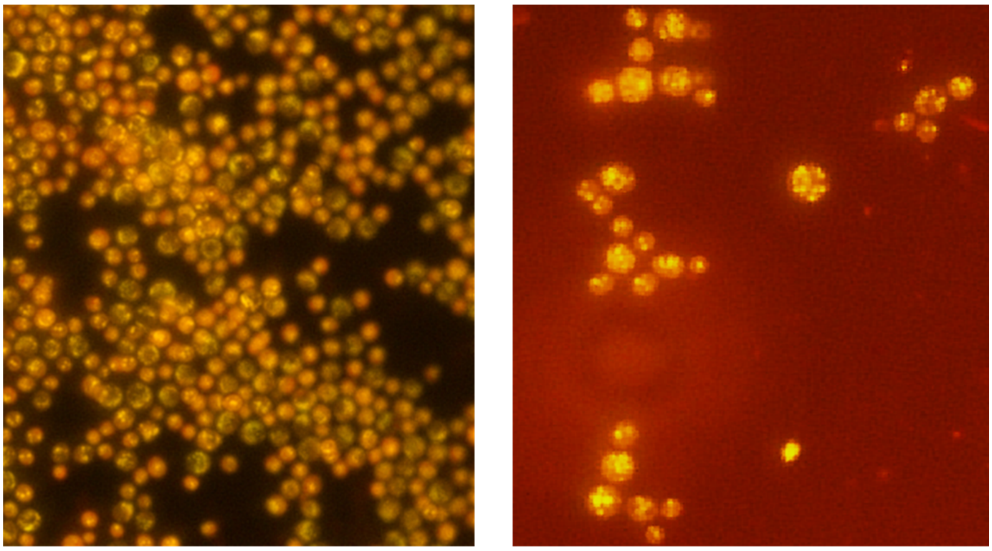

WS **FES strain**

Supplementary figure.4. Nile red staining dye was used to visualize the lipid droplets biogenesis and accumulation of squalene in engineered strain FSE compared with wild strain.

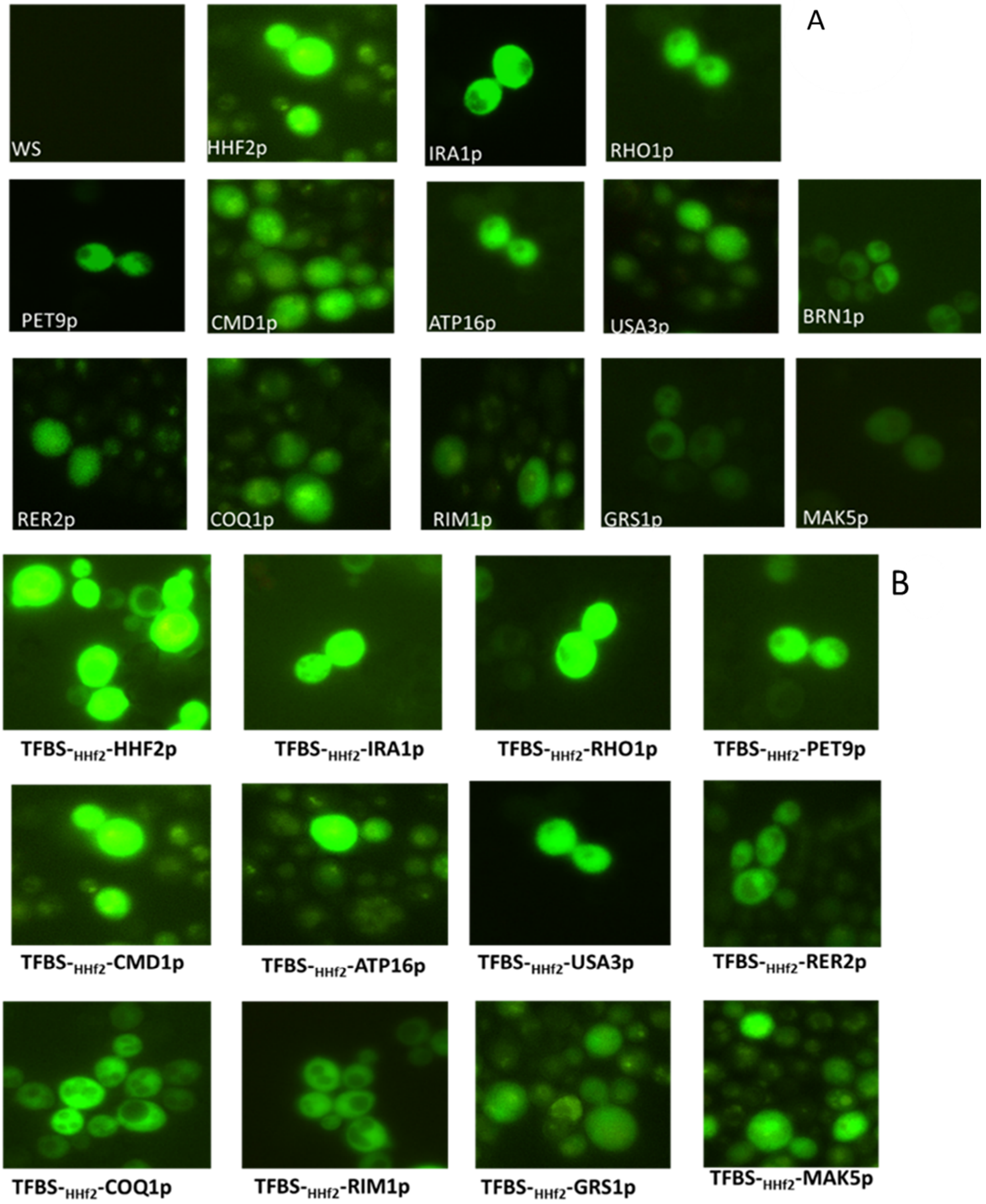
A
WS
HHF2p
IRA1p
RHO1p
PET9p
CMD1p
ATP16p
USA3p
BRN1p
RER2p
COQ1p
RIM1p
GRS1p
MAK5p
B
TFBS-$_{HHf2}$-HHF2p
TFBS-$_{HHf2}$-IRA1p
TFBS-$_{HHf2}$-RHO1p
TFBS-$_{HHf2}$-PET9p
TFBS-$_{HHf2}$-CMD1p
TFBS-$_{HHf2}$-ATP16p
TFBS-$_{HHf2}$-USA3p
TFBS-$_{HHf2}$-RER2p
TFBS-$_{HHf2}$-COQ1p
TFBS-$_{HHf2}$-RIM1p
TFBS-$_{HHf2}$-GRS1p
TFBS-$_{HHf2}$-MAK5p

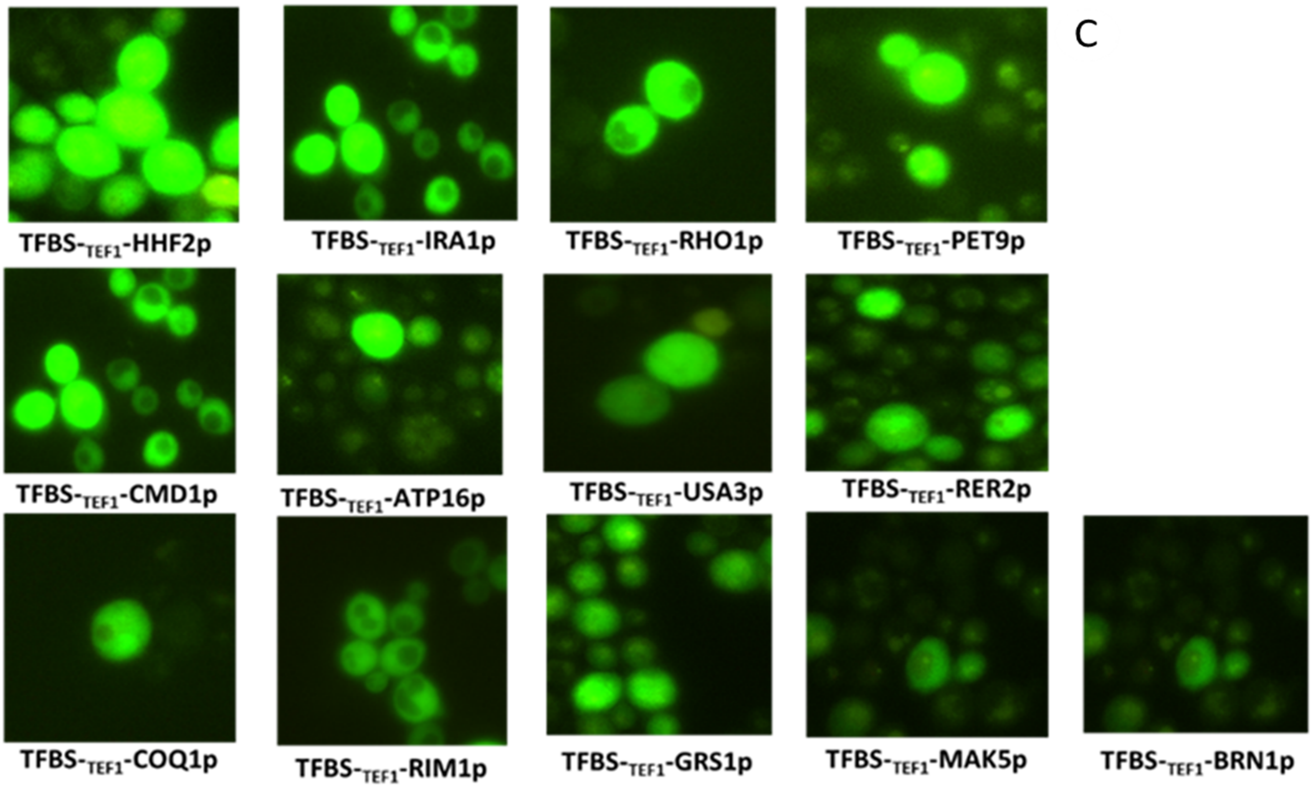

Supplementary figure.5. (a) Confocal microscopic analysis of wild type yeast promoters. (b) Confocal microscopic analysis of engineered promoters containing TFBS of HHF2p. The attachment of TFBS of HHF2p enhanced the strength of all promoters. (c) Confocal microscopic analysis of engineered promoters containing TFBS of TEF12p. The attachment of TFBS of TEF1p enhanced the strength of all promoters.

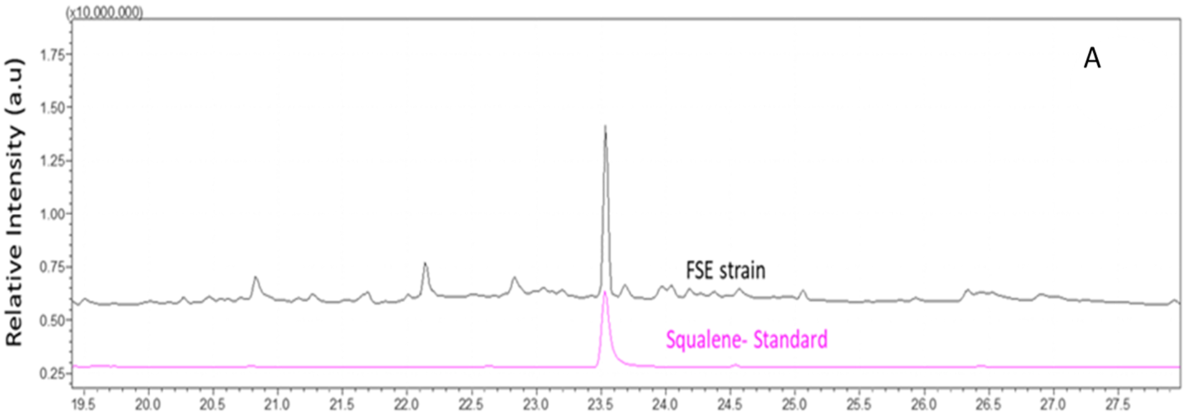

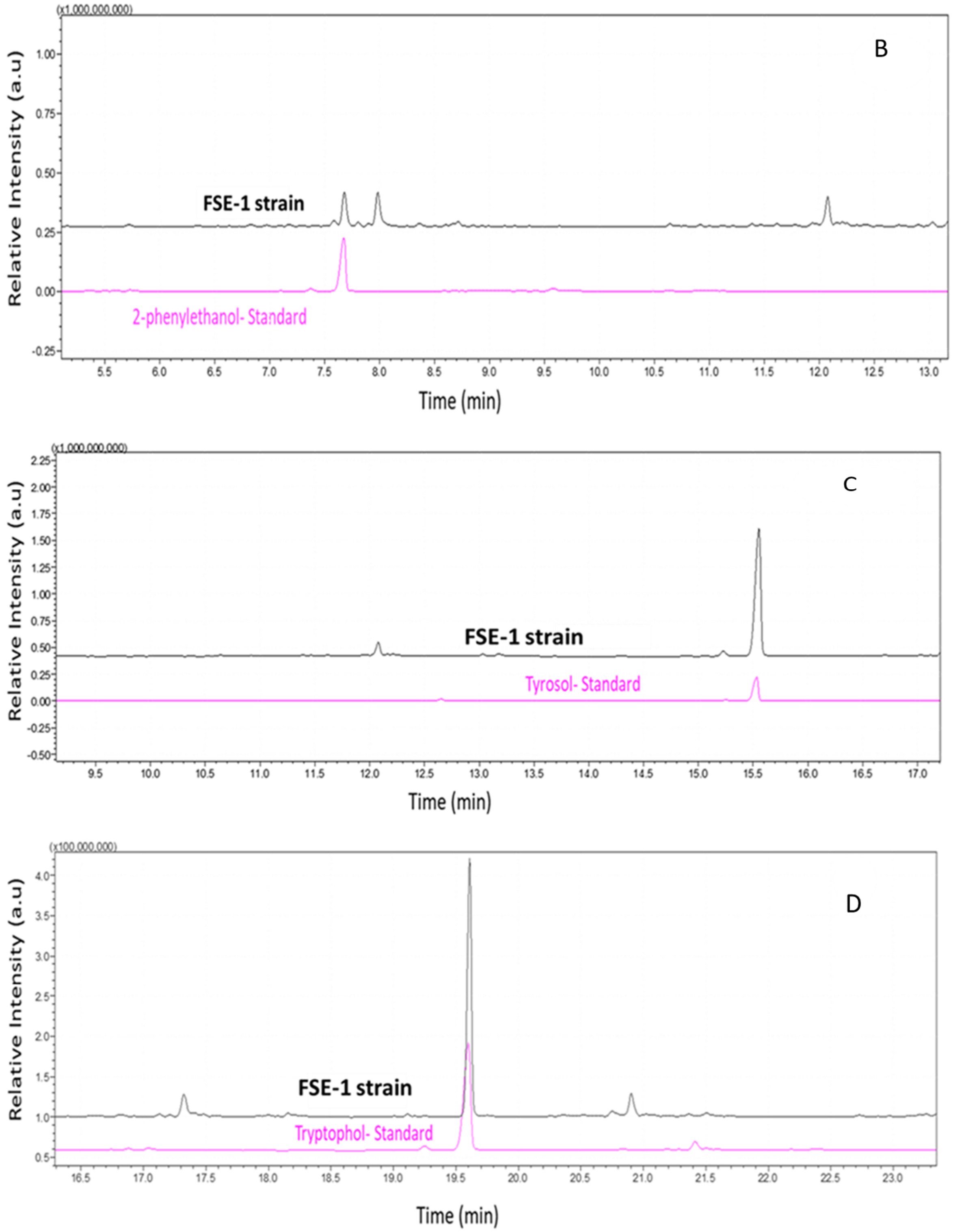

B
(x1,000,000,000)
Relative Intensity (a.u)
FSE-1 strain
2-phenylethanol- Standard
Time (min)
C
(x1,000,000,000)
Relative Intensity (a.u)
FSE-1 strain
Tyrosol- Standard
Time (min)
D
(x100,000,000)
Relative Intensity (a.u)
FSE-1 strain
Tryptophol- Standard
Time (min)

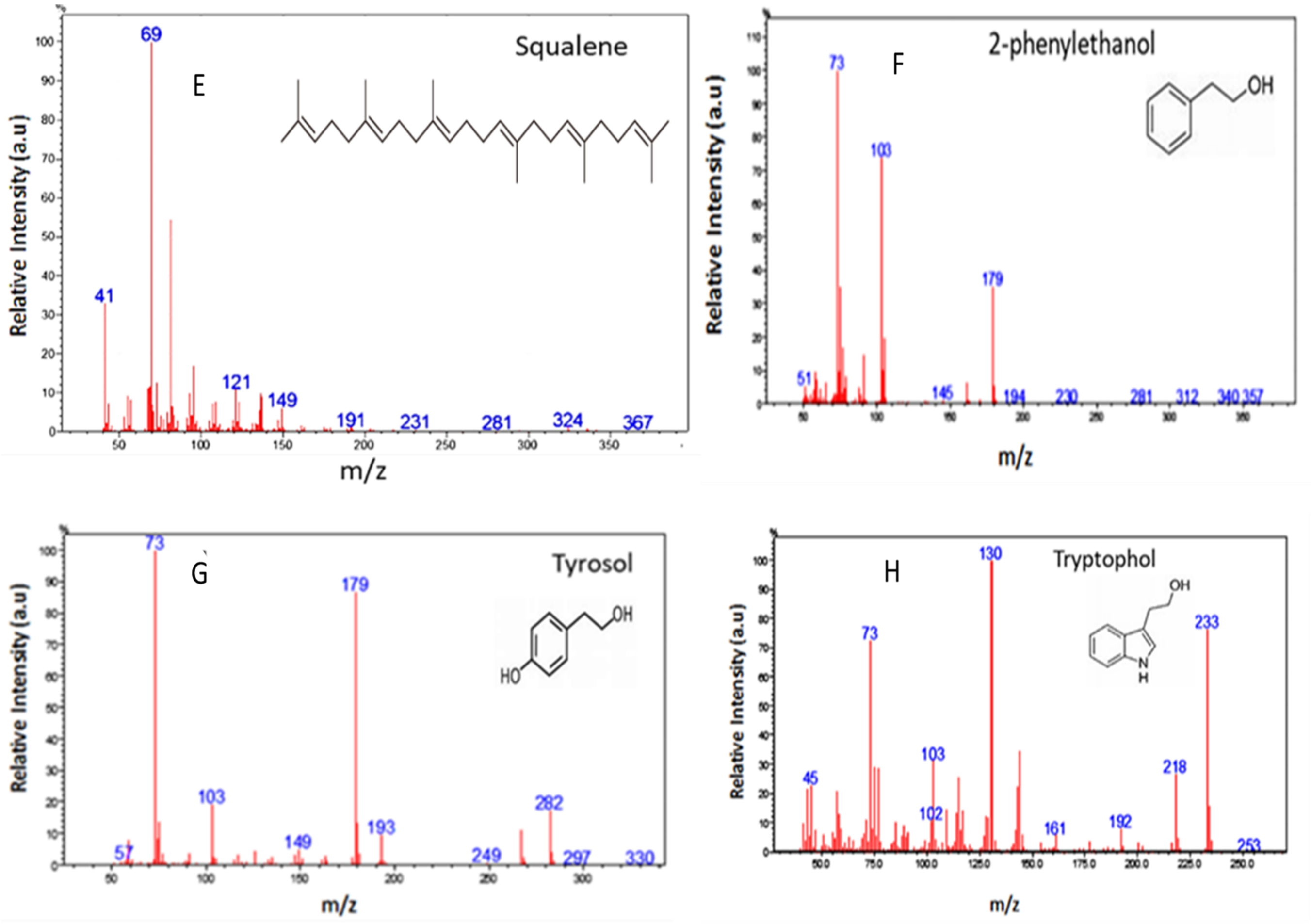

Supplementary figure. S6. GCMS analysis of metabolites: (a) GC spectrogram of squalene. The production of squalene in FSE strain was confirmed and quantified by comparing the spectrogram of sample and authentic standard of squalene. (**b**) GC spectrogram of 2-phenylethanol. The production of 2-phenylethanol in FSE1 strain was confirmed and quantified by comparing the spectrogram of sample and authentic standard of 2-phenylethanol. (**c**) GC spectrogram of tyrosol. The production of tyrosol in FSE1 strain was confirmed and quantified by comparing the spectrogram of sample and authentic standard of tyrosol. (**d**) GC spectrogram of tryptophol. The production of tryptophol in FSE1 strain was confirmed and quantified by comparing the spectrogram of sample and authentic standard of tryptophol. (e) MS spectrogram of squalene. The production of squalene in FSE strain was also verified by matching the fragmentation pattern of squalene in sample with the database of NIST archive. (**f**) MS spectrogram of 2-phenylethanol. The production of 2-phenylethanol in FSE1 strain was also verified by matching the fragmentation pattern of 2-phenylethanol in sample with the database of NIST archive. (**g**) MS spectrogram of tyrosol. The production of tyrosol in FSE1 strain was also verified by matching the fragmentation pattern of tyrosol in sample with the database of NIST archive. (h) MS spectrogram of tryptophol. The production of tryptophol in FSE1 strain was also verified by matching the fragmentation pattern of tryptophol in sample with the database of NIST archive.
